# Author Correction: Expansion of human megakaryocyte-biased hematopoietic stem cells by biomimetic Microniche

**DOI:** 10.1038/s41467-025-60584-w

**Published:** 2025-06-05

**Authors:** Yinghui Li, Mei He, Wenshan Zhang, Wei Liu, Hui Xu, Ming Yang, Hexiao Zhang, Haiwei Liang, Wenjing Li, Zhaozhao Wu, Weichao Fu, Shiqi Xu, Xiaolei Liu, Sibin Fan, Liwei Zhou, Chaoqun Wang, Lele Zhang, Yafang Li, Jiali Gu, Jingjing Yin, Yiran Zhang, Yonghui Xia, Xuemei Mao, Tao Cheng, Jun Shi, Yanan Du, Yingdai Gao

**Affiliations:** 1 State Key Laboratory of Experimental Hematology, National Clinical Research Center for Blood Diseases, Haihe Laboratory of Cell Ecosystem, PUMC Department of Stem Cell and Regenerative Medicine, CAMS Key Laboratory of Gene Therapy for Blood Diseases, Institute of Hematology and Blood Diseases Hospital, Chinese Academy of Medical Sciences & Peking Union Medical College, Tianjin, 300020 China; 2 Tianjin Institutes of Health Science, Tianjin, 301600 China; 3 Department of Biomedical Engineering, School of Medicine, Tsinghua-PKU Center for Life Sciences, Tsinghua University, 100084 Beijing, China; 4 Beijing CytoNiche Biotechnology Co. Ltd., 100195 Beijing, China; 5 Nankai Hospital, Tianjin Hospital of Integrated Traditional Chinese and Western Medicine, Tianjin, 300100 China

**Keywords:** Haematopoietic stem cells, Stem-cell biotechnology, Haematopoiesis

Correction to: *Nature Communications* 10.1038/s41467-023-37954-3, published online 18 April 2023

In the version of the article initially published, due to a preparation error, a flow cytometry plot in Fig. 1c (bottom row, center) was mistakenly a duplicate of Fig. 4e, while a plot in Fig. 1d (bottom row, right-hand side) was a duplicate of Supplementary Fig. 5f. Figure 1 has now been amended in the HTML and PDF versions of the article.

