# Supplementary material for: Expansion of human megakaryocyte-biased hematopoietic stem cells by biomimetic Microniche
Source: Nat Commun. 2023 Apr 18;14:2207. doi: 10.1038/s41467-023-37954-3 (PMC10113370; doi:10.1038/s41467-023-37954-3)
Supplement: Supplementary file 1 — Supplementary Information [file 41467_2023_37954_MOESM1_ESM.pdf]

SUPPLEMENTARY INFORMATION

Expansion of Human Megakaryocyte-biased Hematopoietic Stem Cells by Biomimetic Microniche

Yinghui Li et al.

Figure S1

a

Microniche Tablets	
Appearance	White or slight yellow thin disc
Weight	20 mg/tablet
Particle Size	165±10 μm
D5-D95	100-220 μm
Pore size	25±5 μm
Surface area	12000 cm^2/g
PH	7.0±0.5
Particles per gram (dry weight)	5.0*10^6
Absorptivity	About 15 uL/mg
Dispersibility	Dispersible into colloidal solution in 20 s
Swelling factor	20-30 ml/g
Degradability*	Fully degraded in 15minutes at 37°C
Sterility	Sterile
Mycoplasma	No mycoplasma
Endotoxin	Less than 0.5 EU/mg

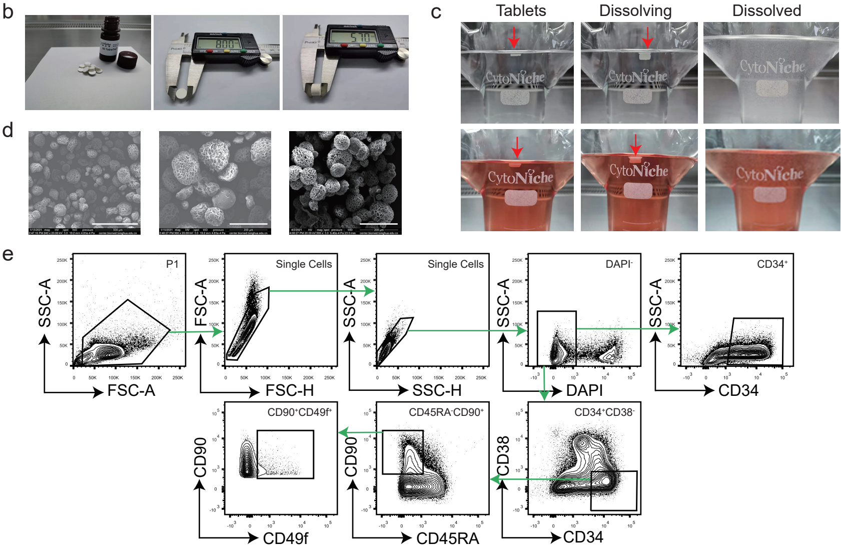

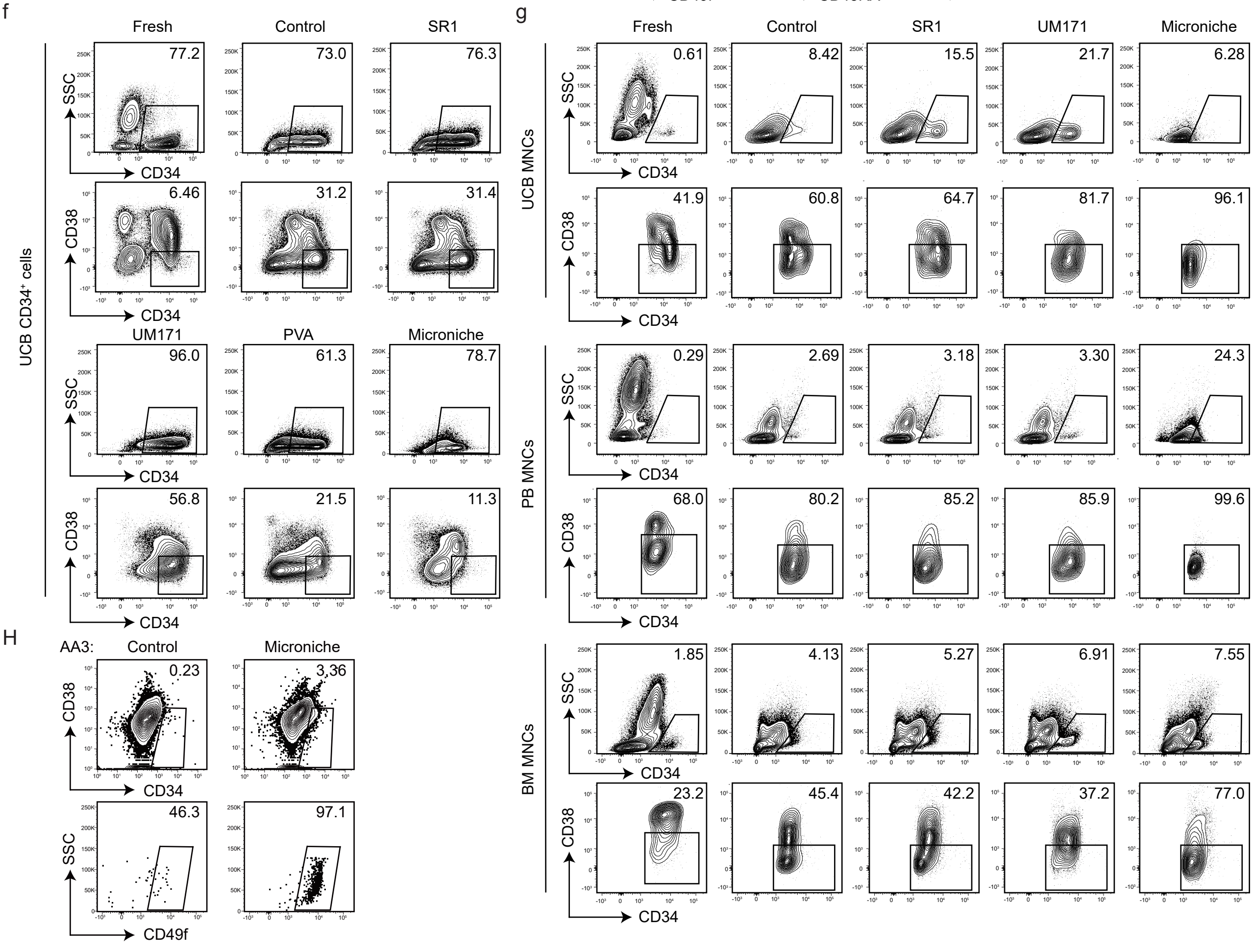

Supplementary Figure 1. Characters of Microniche and *in vitro* culture data with Microniche. a Physical parameters of Microniche tablets. *Digested by 3D FloTrix® Digest. **b** Appearance and size of Microniche tablets. The diameter and thickness of the tablets were measured using an electronic digital caliper. The graphs represent a mean of three independent mesurements (10 replicates analysed in each). **c** Solubility diagram of Microniche tablets in PBS and cell culture medium. **d** Scanning electron microscope images of Microniche. Scale bar, 500 μm (left) and 200 μm (middle and right). **e** Flow cytometry gating strategies for HSPC and and HSC subpopulations during *in vitro* culture. **f** Representative FACS profiles of $CD34^+$ and $CD34^+CD38^-$ populations in UCB $CD34^+$ cells before (fresh) and after cultured in control medium or control medium supplemented with SR1 (1μM), UM171 (40nM), PVA (0.1%) or Microniche. Fresh, n = 3 technical replicates; Microniche, n = 3 biological replicates; Other groups, n = 4 biological replicates. **g** Representative FACS profiles of $CD34^+$ and $CD34^+CD38^-$ populations in UCB, PB or BM MNCs before (fresh) and after culture in control medium, or control medium supplemented with SR1 (1μM), UM171 (40nM) or Microniche. For BM and PB MNCs, n = 3 biological replicates; for UCB MNCs, fresh n = 4 technical replicates and other groups n = 3 biological replicates. **h** Representative FACS profiles of $CD34^+CD38^-$ and $CD34^+CD38^-CD49f^+$ populations derived from AA patient 3 after culture.

Figure S2

a

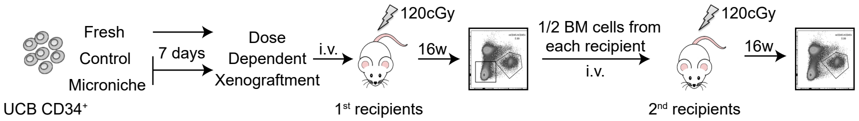

b

Treatment	Dose of starting CD34⁺ cells	Tested	1st Engrafted (2nd Tested)	2nd Engrafted	1st Stem Cell Frequency and *P*-value	2nd Stem Cell Frequency and *P*-value
Fresh	100000	4	4	4	1/11994	1/51557
	50000	4	3	0		
	10000	6	4	2		
	1000	11	2	0		
	100	12	2	1		
	10	6	0	0		
Control	50000	2	1	0	1/16624<br>(vs Fresh) 0.57	1/116929<br>(vs Fresh) 0.405
	10000	2	2	1		
	2000	4	2	0		
	400	8	0	0		
	80	11	0	0		
	16	3	0	0		
Microniche	100000	2	2	1	1/2394<br>(vs Fresh) 0.00172<br>(vs Control) 0.000804	1/68000<br>(vs Fresh) 0.67<br>(vs Control) 0.612
	20000	6	6	2		
	4000	5	4	1		
	800	12	3	0		
	160	5	0	0		
	32	6	1	0		

c

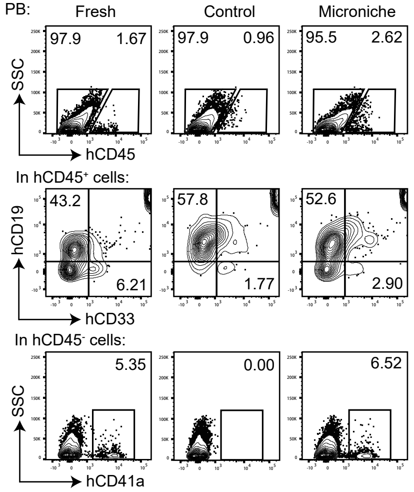

d

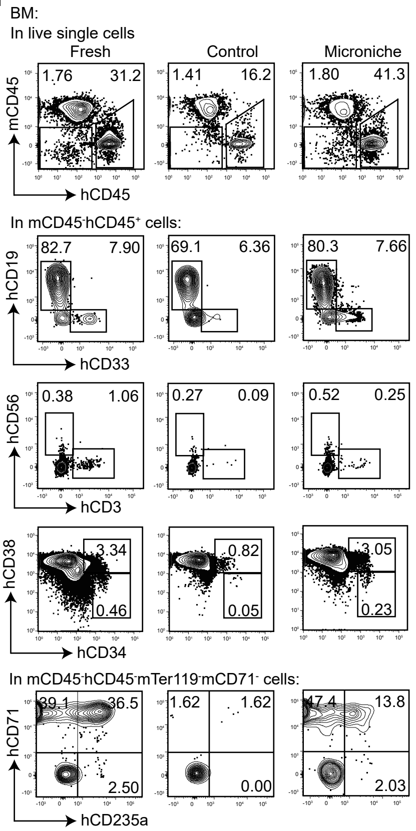

e

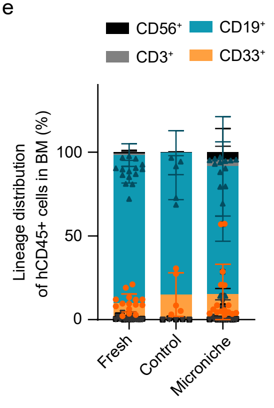

f

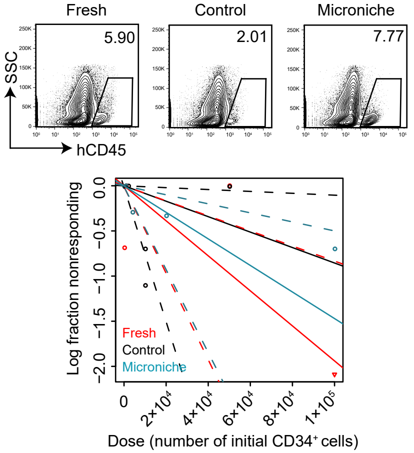

Supplementary Figure 2. *Ex vivo* culture data with Microniche. a Schematic representation of the LDA experiment design. NOG mice were transplanted with different day 0 equivalent cell doses of fresh (fresh) UCB CD34$^+$ cells or their progeny cultured with or without Microniche for 7 days. **b** Limit dilution analysis of NOG engraftment at 16 weeks post-transplantation. The table summarizing primary and secondary engraftment and stem cell frequency. If an obvious positive cell cluster was observed, the mice were considered to be successfully engrafted regardless of the portion of hCD45$^+$. Stem cell frequency analyzed by Chi-square test. **c** Representative FACS profiles of the engrafting human CD45$^+$ cells in DAPI$^-$ cells, human myeloid (hCD33$^+$) and B-lymphoid (hCD19$^+$) cells in hCD45$^+$ cells, human megakaryocytes (hCD41a$^+$) in hCD45$^-$ cells in PB of primary NOG mice 16 weeks post-transplantation. **d** Representative FACS profiles of the engrafting human CD45$^+$ cells and multilineage reconstitution in BM of primary NOG mice 16 weeks post-transplantation. Human myeloid (hCD33$^+$), B-lymphoid (hCD19$^+$), T-lymphoid (hCD3$^+$), natural killer cells (NK, hCD56$^+$), hematopoietic stem (hCD34$^+$CD38$^-$) and progenitor (hCD34$^+$CD38$^+$) cells in mCD45$^-$hCD45$^+$ cells and erythroid lineage (hCD71$^+$CD235a$^-$, hCD71$^+$CD235a$^+$, hCD71$^-$CD235a$^+$) in mCD45$^-$hCD45$^-$mTer119$^-$ mCD71$^-$ cells are shown. **e** Relative distribution of myeloid, B-lymphoid, T-lymphoid and NK cells as a percentage of hCD45$^+$ cells in BM of primary engrafted NOG mice. Fresh n = 15, Control n = 5, Microniche n = 16 biologically independent animals. Data are means ± s.d. and no significant difference between groups. **f** Representative FACS profiles of the engrafting human CD45$^+$ cells and graph of limit dilution analysis of secondary engraftment. Chi-square test. Solid lines indicate best-fit linear model and dashed lines confidence intervals.

Figure S3

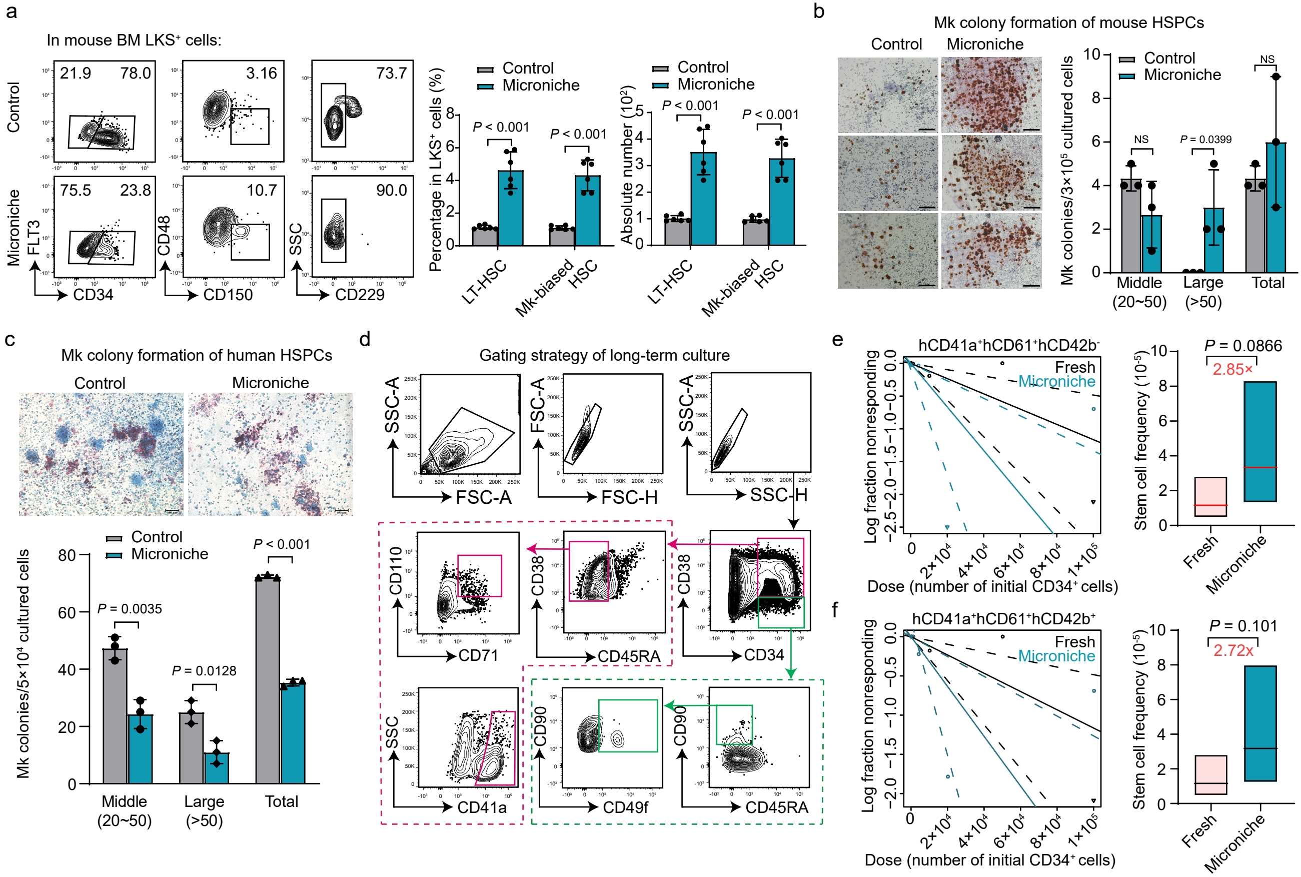

a
In mouse BM LKS+ cells:
Control
Microniche
FLT3
CD34
CD48
CD150
SSC
CD229
21.9
78.0
3.16
73.7
75.5
23.8
10.7
90.0
Control
Microniche
Percentage in LKS+ cells (%)
P < 0.001
P < 0.001
LT-HSC
Mk-biased HSC
Absolute number (10^2)
b
Mk colony formation of mouse HSPCs
Control
Microniche
Mk colonies/3×10^5 cultured cells
NS
P = 0.0399
NS
Middle (20~50)
Large (>50)
Total
c
Mk colony formation of human HSPCs
Control
Microniche
Mk colonies/5×10^4 cultured cells
P = 0.0035
P = 0.0128
P < 0.001
Middle (20~50)
Large (>50)
Total
d
Gating strategy of long-term culture
SSC-A
FSC-A
FSC-H
SSC-H
CD110
CD71
CD38
CD45RA
CD34
SSC
CD41a
CD90
CD49f
CD45RA
e
hCD41a+hCD61+hCD42b-
Fresh
Microniche
Log fraction nonresponding
Dose (number of initial CD34+ cells)
Stem cell frequency (10^-5)
P = 0.0866
2.85×
Fresh
Microniche
f
hCD41a+hCD61+hCD42b+
Fresh
Microniche
Log fraction nonresponding
Dose (number of initial CD34+ cells)
Stem cell frequency (10^-5)
P = 0.101
2.72x
Fresh
Microniche

Supplementary Figure 3. Effect of Microniche on Mk lineage. a Representative FACS profiles of $LKS^{+}CD34^{-}CD150^{+}CD48^{-}$ (LT-HSC) and $LKS^{+}CD34^{-}CD150^{+}CD48^{-}CD229^{-}$ (Mk-biased HSC) in cultured (day 7) mice c-Kit^{+} cells. The percentage and the absolute number of LT-HSC and Mk-biased HSC are shown (n = 6 biological replicates). All data represent means ± s.d.; unpaired two-tailed Student's t-test. **b** Formation of mouse megakaryocytic colonies (CFU-Mk) in a collagen-based culture system (n = 3 biological replicates). All data represent means ± s.d.; unpaired two-tailed Student's t-test. Representative morphology images are also shown. Scale bars, 100 µm. Colony size: middle (20 to 50 cells), or large (>50 cells). **c** Human CFU-Mk (n = 3 biological replicates). All data represent means ± s.d.; unpaired two-tailed Student's t-test. Representative morphology images are also shown. Scale bars, 100 µm. **d** Flow cytometry gating strategies for HSC and and Mk subpopulations during long-term culture *in vitro*. **e-f** Graphs and boxplots of Mk-HSC frequencies based on phenotype-defined subpopulations $mCD45^{-}hCD45^{-}mCD41^{-}mCD42d^{-}mCD61^{-}hCD41a^{+}hCD61^{+}hCD42b^{-}$ (**e**) and $CD45^{-}hCD45^{-}mCD41^{-}mCD42d^{-}mCD61^{-}hCD41a^{+}hCD61^{+}hCD42b^{+}$ (**f**) 16 weeks after transplantation. Fresh n = 43, Control n = 30, Microniche n = 36 biologically independent animals. Chi-square test. Solid lines indicate best-fit linear model and dashed lines confidence intervals. Box plots show the median (middle line) with the 25th and 75th percentiles (box).

Figure S4

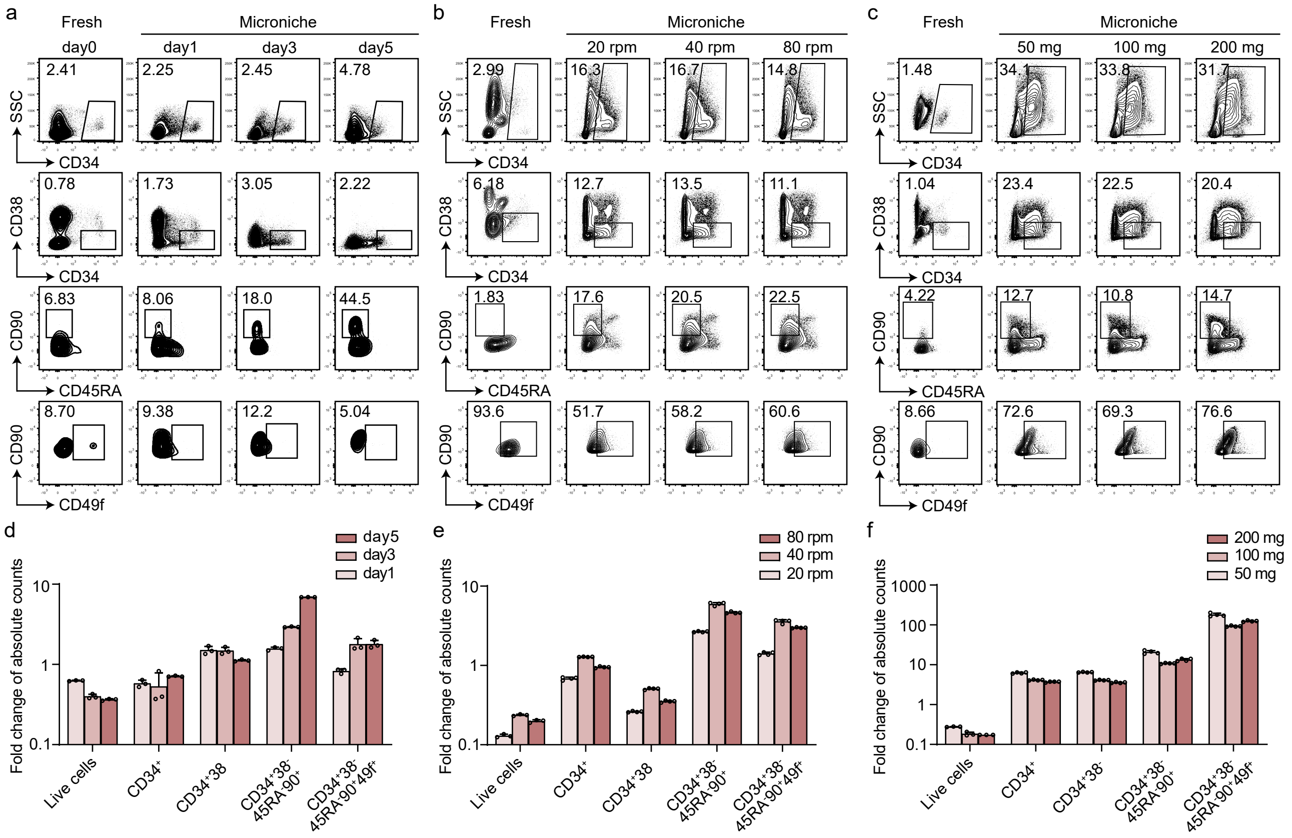

g

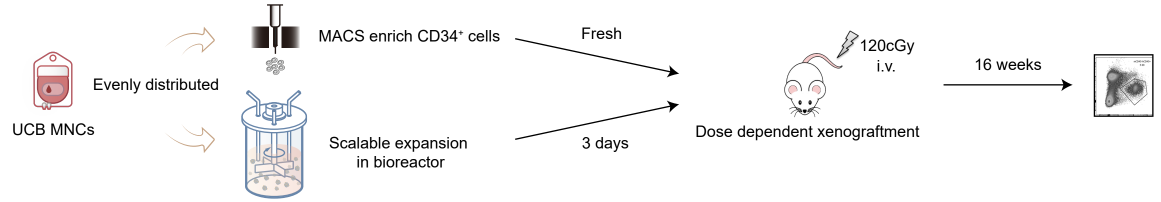

h

Treatment	Dose of starting MNCs	Dose of CD34⁺ cells	Tested	hCD45⁺ Engrafted	HSC frequency and *P*-value	hCD41a⁺ Engrafted	Mk-biased HSC frequency and *P*-value
Fresh	6430000	22260	12	9	1/1869794	2	1/8246664
	643000	2226	7	5		2	
	64300	222.6	8	5		3	
	6430	22.26	6	1		2	
Microniche	6430000	-	10	10	1/253087 (vs Fresh) 0.000176	9	1/1453922 (vs Fresh) 0.000102
	643000	-	12	10		7	
	64300	-	9	3		1	
	6430	-	9	2		0	

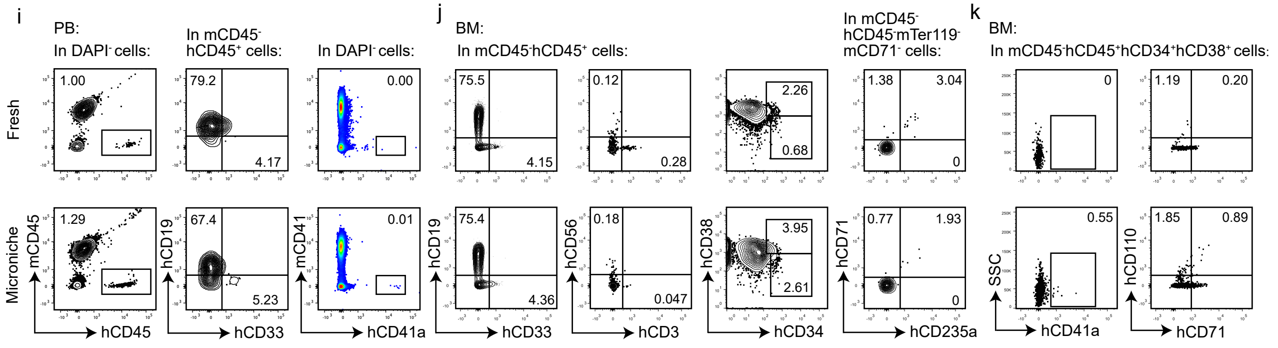

Supplementary Figure 4. Large-scale dynamic culture condition screening. a-c Representative FACS profiles of $CD34^+$, $CD34^+CD38^-$, $CD34^+CD38^-CD45RA^-CD90^+$, and $CD34^+CD38^-CD45RA^-CD90^+CD49f^+$ populations in UCB MNCs before (fresh = day 0, n = 3 technical replicates) and after cultured with the Microniche for different days (n = 3 biological replicates) (**a**), reactor speeds (n = 4 biological replicates) (**b**) and Microniche doses (n = 4 biological replicates) (**c**) in a dynamic stirred bioreactor. **d-f** Statistics of fold changes in absolute counts of the indicated cell populations at different time points (**d**), reactor speeds (**e**) and Microniche doses (**f**) compared to fresh. Fresh, n = 3 technical replicates; Microniche, n = 3 or 4 biological replicates. All data represent means ± s.d. **g** Schematic representation of the LDA experiment design of bulk-culture. **h** Limit dilution analysis of NCG engraftment at 16 weeks post-transplantation. The table summarizing $hCD45^+$ and $hCD41a^+$ engraftment, and stem cell frequency. If no less than 0.01% $mCD45^-hCD45^+$ cells or no less than 0.01% hCD41a+ cells in $mCD45^-hCD45^-mCD41^-mCD42d^-mCD62^-$ population were observed, the mice were considered to be successfully engrafted or Mk reconstructed. HSC and Mk-biased HSC frequencies analyzed by Chi-square test. **i** Representative FACS profiles of the engrafting human $CD45^+$ cells in $DAPI^-$ $mCD45^-$ cells, human myeloid ($hCD33^+$) and B-lymphoid ($hCD19^+$) cells in $mCD45^-hCD45^+$ cells, human megakaryocytes ($hCD41a^+$) in $DAPI^-mCD41^-$ cells in PB of primary NOG mice 8 weeks post-transplantation. **j** Representative FACS profiles of the multilineage reconstitution in BM of NCG mice 16 weeks post-transplantation. Human myeloid ($hCD33^+$), B-lymphoid ($hCD19^+$), T-lymphoid ($hCD3^+$), natural killer cells (NK, $hCD56^+$), hematopoietic stem ($hCD34^+CD38^-$) and progenitor ($hCD34^+CD38^+$) cells in $mCD45^-hCD45^+$ cells and erythroid lineage ($hCD71^+CD235a^-$, $hCD71^+CD235a^+$, $hCD71^-CD235a^+$) in $mCD45^-hCD45^-mTer119^-$ $mCD71^-$ cells are shown. **k** Representative FACS profiles of Mk reconstruction in BM $mCD45^-$ $hCD45^+hCD34^+hCD38^+$ cells of NCG mice at 16 weeks post-transplantation.

Figure S5

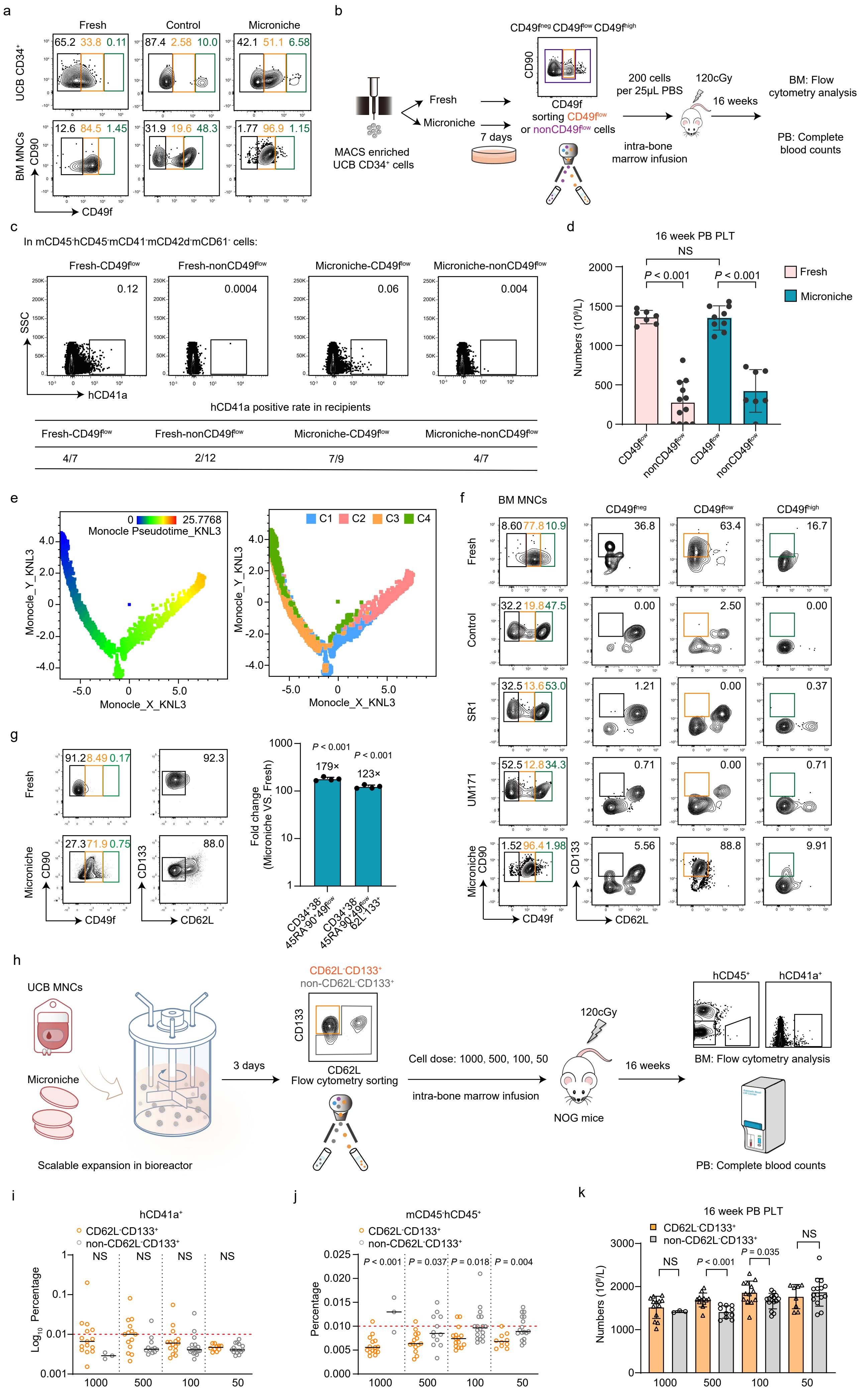
a
Fresh
Control
Microniche
UCB CD34+
BM MNCs
CD90
CD49f
65.2 33.8 0.11
87.4 2.58 10.0
42.1 51.1 6.58
12.6 84.5 1.45
31.9 19.6 48.3
1.77 96.9 1.15
b
MACS enriched
UCB CD34+ cells
Fresh
Microniche
7 days
CD49fneg CD49flow CD49fhigh
CD90
CD49f
sorting CD49flow
or nonCD49flow cells
200 cells
per 25μL PBS
intra-bone
marrow infusion
120cGy
16 weeks
BM: Flow
cytometry analysis
PB: Complete
blood counts
c
In mCD45-hCD45+mCD41-mCD42d-mCD61- cells:
Fresh-CD49flow
Fresh-nonCD49flow
Microniche-CD49flow
Microniche-nonCD49flow
0.12
0.0004
0.06
0.004
SSC
hCD41a
hCD41a positive rate in recipients
Fresh-CD49flow
Fresh-nonCD49flow
Microniche-CD49flow
Microniche-nonCD49flow
4/7
2/12
7/9
4/7
d
16 week PB PLT
NS
P < 0.001
P < 0.001
Fresh
Microniche
Numbers (10^9/L)
CD49flow
nonCD49flow
CD49flow
nonCD49flow
e
0
25.7768
Monocle Pseudotime_KNL3
Monocle_Y_KNL3
Monocle_X_KNL3
C1
C2
C3
C4
f
BM MNCs
CD49fneg
CD49flow
CD49fhigh
Fresh
Control
SR1
UM171
Microniche
8.60 77.8 10.9
36.8
63.4
16.7
32.2 19.8 47.5
0.00
2.50
0.00
32.5 13.6 53.0
1.21
0.00
0.37
52.5 12.8 34.3
0.71
0.00
0.71
1.52 96.4 1.98
5.56
88.8
9.91
CD90
CD49f
CD133
CD62L
g
Fresh
Microniche
91.2 8.49 0.17
92.3
27.3 71.9 0.75
88.0
CD90
CD49f
CD133
CD62L
P < 0.001
P < 0.001
179×
123×
Fold change
(Microniche VS. Fresh)
CD34+38-45RA-90+49flow
CD34+38-45RA-90+49flow62L-133+
h
UCB MNCs
Microniche
Scalable expansion in bioreactor
3 days
CD62L-CD133+
non-CD62L-CD133+
CD133
CD62L
Flow cytometry sorting
Cell dose: 1000, 500, 100, 50
intra-bone marrow infusion
120cGy
NOG mice
16 weeks
hCD45+
hCD41a+
BM: Flow cytometry analysis
PB: Complete blood counts
i
hCD41a+
CD62L-CD133+
non-CD62L-CD133+
Log10 Percentage
NS
NS
NS
NS
1000
500
100
50
j
mCD45-hCD45+
CD62L-CD133+
non-CD62L-CD133+
Percentage
P < 0.001
P = 0.037
P = 0.018
P = 0.004
1000
500
100
50
k
16 week PB PLT
CD62L-CD133+
non-CD62L-CD133+
Numbers (10^9/L)
NS
P < 0.001
P = 0.035
NS
1000
500
100
50

Supplementary Figure 5. Special subpopulation maintained by Microniche. a Representative FACS profiles showing CD49f distribution in UCB CD34$^+$ cells and BM MNCs before (fresh) and after culture in Microniche or control generated by gating CD34$^+$CD38$^-$CD45RA$^-$CD90$^+$ cells. **b** Experimental diagram of xenograft using CD49f^{low} and nonCD49f^{low} cells from CD34$^+$CD38$^-$CD45RA$^-$CD90$^+$. **c** Upper panel: Representative FACS profiles of Mk reconstitution in BM mCD45$^-$hCD45$^-$mCD41$^-$mCD42d$^-$mCD61$^-$ cells of NOG mice 16 weeks post-transplantation with sorted CD49f^{low} (CD34$^+$CD38$^-$CD45RA$^-$CD90$^+$CD49f^{low}) or nonCD49f^{low} (CD34$^+$CD38$^-$CD45RA$^-$CD90$^+$CD49f$^{neg\&high}$ cells). Lower panel: The proportion of successful reconstitution of Mk lineage based on hCD41a expression in BM mCD45$^-$hCD45$^-$mCD41$^-$mCD42d$^-$mCD61$^-$ cells of NOG mice. **d** Platelet (PLT) counts in PB of recipients at week 16 post-transplantation. Fresh-CD49f^{low} *n* = 7, Fresh-nonCD49f^{low} *n* = 12, Microniche-CD49f^{low} *n* = 9, Microniche-nonCD49f^{low} *n* = 7 biologically independent animals. All data represent the means ± s.d.; unpaired two-tailed Students' *t*-test. **e** Pseudotime analysis by Monocle 2 (left). Trajectory analysis combining four cell clusters indicates the developmental path from C2 to C4 (right). **f** Representative FACS profiles of CD62L$^-$CD133$^+$ distribution within CD34$^+$CD38$^-$CD45RA$^-$CD90$^+$CD49f$^{neg/low/high}$ subpopulations of BM MNCs before (fresh) and after 7 days of culture in control or control medium supplemented with SR1 (1μM), UM171 (40nM) or Microniche. **g** Representative FACS profiles and fold change of absolute counts of UCB MNC CD34$^+$CD38$^-$CD45RA$^-$CD90$^+$CD49f^{low} and CD34$^+$CD38$^-$CD45RA$^-$CD90$^+$CD49f^{low}CD62L$^-$CD133$^+$ subpopulations before (fresh) and after 3 days of expansion in a Microniche-based bioreactor. Fresh, *n* = 4 technical replicates; Microniche, *n* = 4 biological replicates. All data represent means ± s.d.; unpaired two-tailed Students' *t*-test. **h** Experimental diagram of xenograft using sorted CD62L$^-$CD133$^+$ and non-CD62L$^-$CD133$^+$ (in CD34$^+$CD38$^-$CD45RA$^-$CD90$^+$CD49f^{low}) populations derived from cultured UCB MNCs in a Microniche-based bioreactor. CD62L$^-$CD133$^+$ 1000, 500, 100, 50 group *n* = 15, 14, 15, 10; non-CD62L$^-$CD133$^+$ 1000, 500, 100, 50 group *n* = 3, 12, 18, 17 biologically independent animals. **i** Log percentage level of hCD41a$^+$ cells in mCD45$^-$hCD45$^-$mCD41$^-$mCD42d$^-$mCD61$^-$ population of recipient BM cells. **j** Percentage level of mCD45$^-$hCD45$^+$ cells in recipient BM cells. **k** Platelet (PLT) counts in PB of recipients at week 16 post-transplantation under different doses of transplanted cells. All data represent the means ± s.d.; unpaired two-tailed Students' *t*-test. NS, not significant.

Figure S6

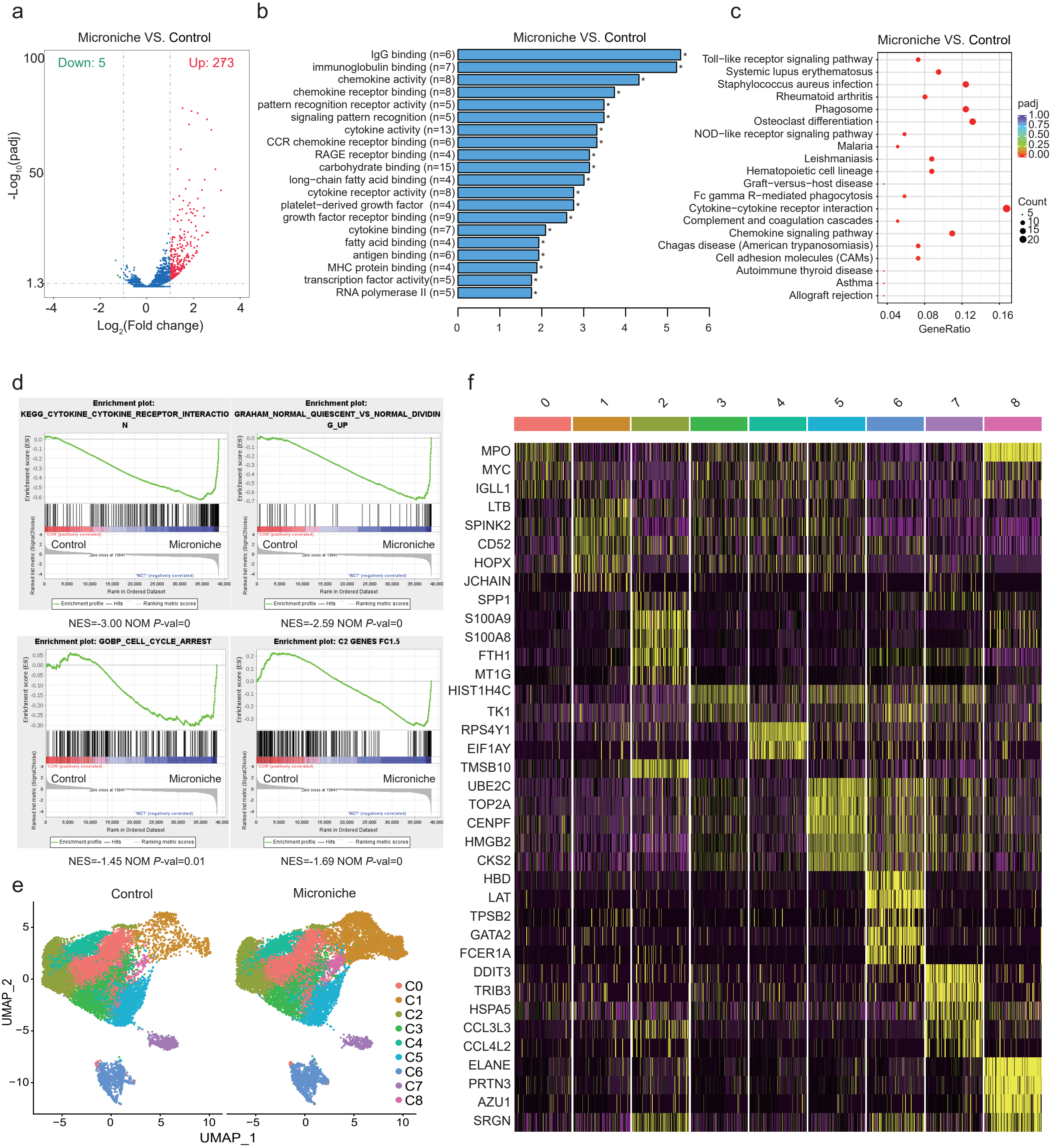
a
Micronice VS. Control
Down: 5
Up: 273
-Log10(padj)
Log2(Fold change)
b
Micronice VS. Control
IgG binding (n=6)
immunoglobulin binding (n=7)
chemokine activity (n=8)
chemokine receptor binding (n=8)
pattern recognition receptor activity (n=5)
signaling pattern recognition (n=5)
cytokine activity (n=13)
CCR chemokine receptor binding (n=6)
RAGE receptor binding (n=4)
carbohydrate binding (n=15)
long−chain fatty acid binding (n=4)
cytokine receptor activity (n=8)
platelet−derived growth factor (n=4)
growth factor receptor binding (n=9)
cytokine binding (n=7)
fatty acid binding (n=4)
antigen binding (n=6)
MHC protein binding (n=4)
transcription factor activity(n=5)
RNA polymerase II (n=5)
c
Micronice VS. Control
Toll−like receptor signaling pathway
Systemic lupus erythematosus
Staphylococcus aureus infection
Rheumatoid arthritis
Phagosome
Osteoclast differentiation
NOD−like receptor signaling pathway
Malaria
Leishmaniasis
Hematopoietic cell lineage
Graft−versus−host disease
Fc gamma R−mediated phagocytosis
Cytokine−cytokine receptor interaction
Complement and coagulation cascades
Chemokine signaling pathway
Chagas disease (American trypanosomiasis)
Cell adhesion molecules (CAMs)
Autoimmune thyroid disease
Asthma
Allograft rejection
padj
Count
GeneRatio
d
Enrichment plot: KEGG_CYTOKINE_CYTOKINE_RECEPTOR_INTERACTION
Enrichment plot: GRAHAM_NORMAL_QUIESCENT_VS_NORMAL_DIVIDING_UP
Control
Micronice
NES=-3.00 NOM P-val=0
NES=-2.59 NOM P-val=0
Enrichment plot: GOBP_CELL_CYCLE_ARREST
Enrichment plot: C2 GENES FC1.5
NES=-1.45 NOM P-val=0.01
NES=-1.69 NOM P-val=0
e
Control
Microniche
UMAP_1
UMAP_2
C0
C1
C2
C3
C4
C5
C6
C7
C8
f
MPO
MYC
IGLL1
LTB
SPINK2
CD52
HOPX
JCHAIN
SPP1
S100A9
S100A8
FTH1
MT1G
HIST1H4C
TK1
RPS4Y1
EIF1AY
TMSB10
UBE2C
TOP2A
CENPF
HMGB2
CKS2
HBD
LAT
TPSB2
GATA2
FCER1A
DDIT3
TRIB3
HSPA5
CCL3L3
CCL4L2
ELANE
PRTN3
AZU1
SRGN

Supplementary Figure 6. Data of RNA sequencing and 10X Genomic single-cell RNA sequencing. a-d, RNA-seq profiling of Microniche cultured UCB CD34$^+$ cells. **a** Volcano plot of differentially expressed genes (DEGs) between Microniche and Control groups. Red, 273 upregulated DEGs; Green, 5 downregulated DEGs. DEGs with the adjusted p-value (padj) <0.05 and $|Log_2(FoldChange)|$ >1. **b** The major GO:MF terms showing enriched expression with padj <0.05 in Microniche-cultured CD34$^+$ cells as indicated in Fig. 5a. **c** KEGG pathways enrichment analysis with padj <0.05. Colors represent padj and bubble size encodes the count of DEGs annotated to the KEGG pathway. GeneRatio represents the proportion of proteins (gene products) in the correspondent abundance groups that is found in each category. **d** GSEA enrichment plots of the gene sets enriched in Microniche versus control, as indicated in Fig. 5b. Genes were ranked using Signal-to-Noise ratio statistics according to their correlation. Vertical black lines mark the position of each gene in the data set. Normalized Enrichment Score (NES) and Nominal *P*-value (NOM *P*-val) are shown. **e** Identification of nine cell clusters in control and Microniche cultures visualized by UMAP in 10X genomics single cell RNA-seq profile of CD34$^+$ cells sorted after culture. Each dot represents one cell, and colours represent distinct cell clusters (C0-C8). **f** Heatmap showing distinct cell clusters (identified by UMAP) annotated by the expression of feature genes in 10X Genomic single-cell RNA sequencing. Relative to Fig. 5e.

Figure S7

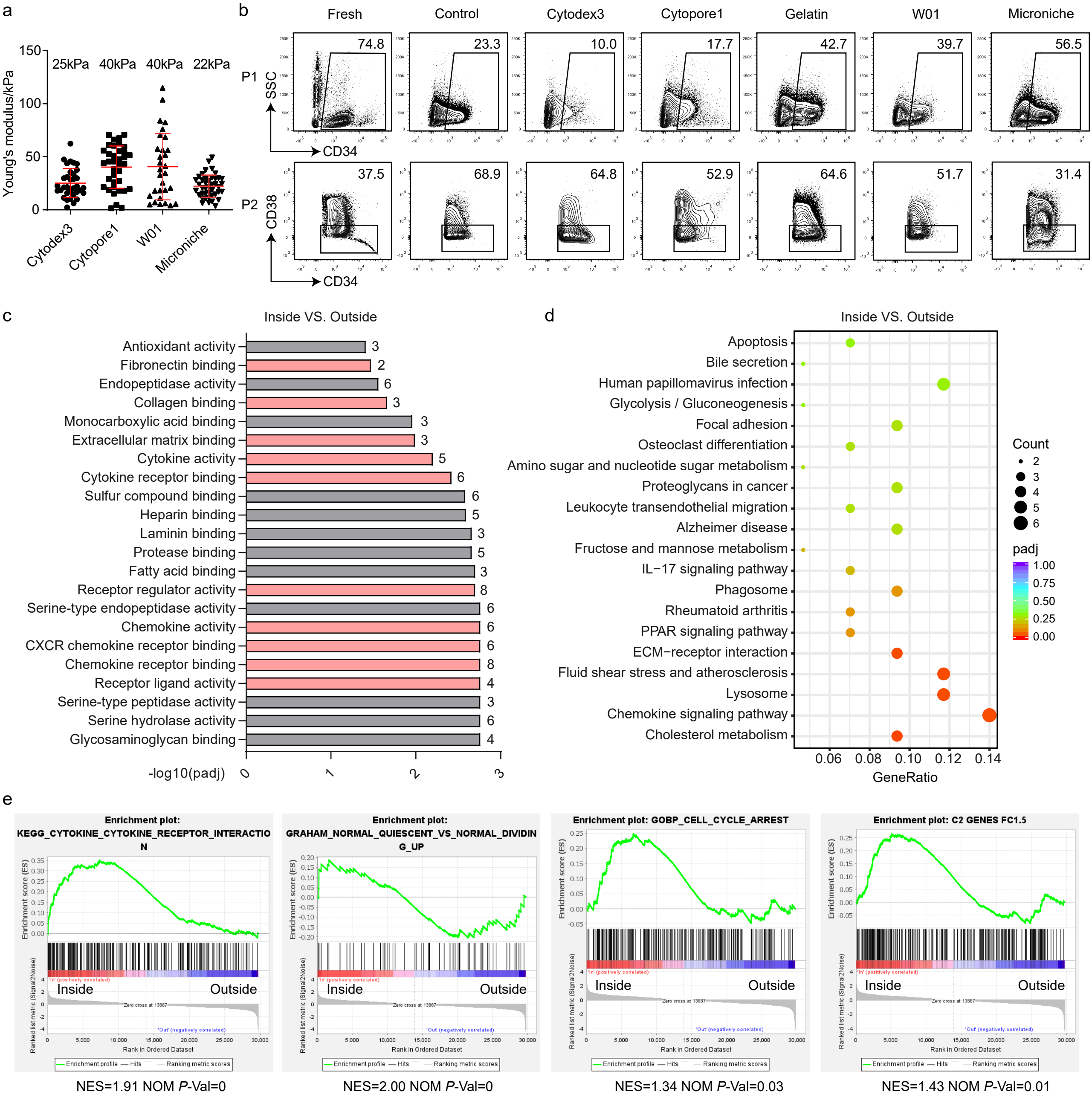
a
Young's modulus/kPa
25kPa
40kPa
40kPa
22kPa
Cytodex3
Cytopore1
W01
Microniche
b
Fresh
Control
Cytodex3
Cytopore1
Gelatin
W01
Microniche
P1
SSC
CD34
74.8
23.3
10.0
17.7
42.7
39.7
56.5
P2
CD38
CD34
37.5
68.9
64.8
52.9
64.6
51.7
31.4
c
Inside VS. Outside
Antioxidant activity 3
Fibronectin binding 2
Endopeptidase activity 6
Collagen binding 3
Monocarboxylic acid binding 3
Extracellular matrix binding 3
Cytokine activity 5
Cytokine receptor binding 6
Sulfur compound binding 6
Heparin binding 5
Laminin binding 3
Protease binding 5
Fatty acid binding 3
Receptor regulator activity 8
Serine-type endopeptidase activity 6
Chemokine activity 6
CXCR chemokine receptor binding 6
Chemokine receptor binding 8
Receptor ligand activity 4
Serine-type peptidase activity 3
Serine hydrolase activity 6
Glycosaminoglycan binding 4
-log10(padj)
d
Inside VS. Outside
Apoptosis
Bile secretion
Human papillomavirus infection
Glycolysis / Gluconeogenesis
Focal adhesion
Osteoclast differentiation
Amino sugar and nucleotide sugar metabolism
Proteoglycans in cancer
Leukocyte transendothelial migration
Alzheimer disease
Fructose and mannose metabolism
IL−17 signaling pathway
Phagosome
Rheumatoid arthritis
PPAR signaling pathway
ECM−receptor interaction
Fluid shear stress and atherosclerosis
Lysosome
Chemokine signaling pathway
Cholesterol metabolism
GeneRatio
Count
padj
e
Enrichment plot: KEGG_CYTOKINE_CYTOKINE_RECEPTOR_INTERACTION
Enrichment plot: GRAHAM_NORMAL_QUIESCENT_VS_NORMAL_DIVIDING_UP
Enrichment plot: GOBP_CELL_CYCLE_ARREST
Enrichment plot: C2 GENES FC1.5
Enrichment score (ES)
Ranked list metric (Signal2Noise)
Rank in Ordered Dataset
Inside
Outside
'In' (positively correlated)
'Out' (negatively correlated)
Zero cross at 13887
Enrichment profile — Hits — Ranking metric scores
NES=1.91 NOM P-Val=0
NES=2.00 NOM P-Val=0
NES=1.34 NOM P-Val=0.03
NES=1.43 NOM P-Val=0.01

Supplementary Figure 7. Data of FACS profiles and RNA sequencing of cultured cells inside and outside Microniche. a The elasticity of different microcarriers based on the Young's modulus data. **b** Representative FACS profiles of $CD34^+$ (P1) and $CD34^+CD38$ (P2) populations in UCB $CD34^+$ cells before (fresh) and after culturing in control medium or control medium supplemented with Cytodex3, Cytopore1, Gelatin, Microcarrier W01, or Microniche. Fresh, *n* = 3 technical replicates; other groups, *n* = 3 biological replicates. **c** The major GO:MF terms showing enriched expression with padj <0.05 in cultured cells inside Microniche as indicated in Figure 6g. **d** KEGG pathways enrichment analysis with padj <0.05. Colours represent padj and bubble size represents the count of DEGs annotated to the KEGG pathway. GeneRatio represents the proportion of proteins (gene products) in the correspondent abundance groups that is found in each category. **e** GSEA enrichment plots of the gene sets enriched in cultured cells inside versus outside the Microniche, as indicated in Fig. 6h. Genes were ranked using Signal-to-Noise ratio statistics according to their correlation. Vertical black lines mark the position of each gene in the data set. Normalized Enrichment Score (NES) and Nominal *P*-value (NOM *P*-val) are shown.

Figure S8

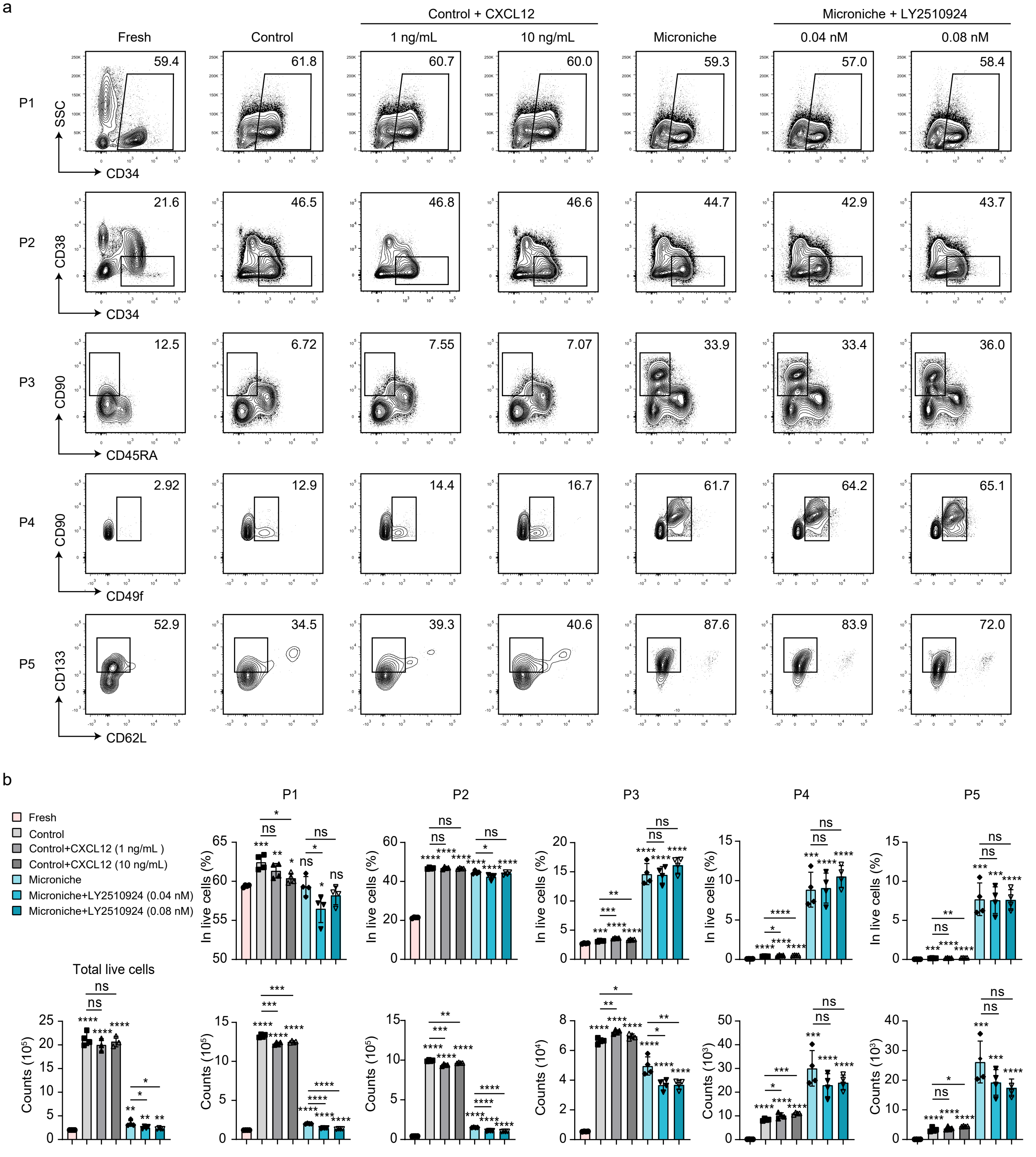

a
Control + CXCL12
Microniche + LY2510924
Fresh
Control
1 ng/mL
10 ng/mL
Microniche
0.04 nM
0.08 nM
P1
SSC
CD34
59.4
61.8
60.7
60.0
59.3
57.0
58.4
P2
CD38
CD34
21.6
46.5
46.8
46.6
44.7
42.9
43.7
P3
CD90
CD45RA
12.5
6.72
7.55
7.07
33.9
33.4
36.0
P4
CD90
CD49f
2.92
12.9
14.4
16.7
61.7
64.2
65.1
P5
CD133
CD62L
52.9
34.5
39.3
40.6
87.6
83.9
72.0
b
Fresh
Control
Control+CXCL12 (1 ng/mL)
Control+CXCL12 (10 ng/mL)
Microniche
Microniche+LY2510924 (0.04 nM)
Microniche+LY2510924 (0.08 nM)
P1
P2
P3
P4
P5
In live cells (%)
Total live cells
Counts (10⁵)
Counts (10⁴)
Counts (10³)

Supplementary Figure 8. The role of CXCR4 in expansion of HSCs by Microniche. a Representative FACS profiles of $CD34^+$ (P1), $CD34^+CD38^-$ (P2), $CD34^+CD38^-CD45RA^-CD90^+$ (P3), $CD34^+CD38^-CD45RA^-CD90^+CD49f^{low}$ (P4), and $CD34^+CD38^-CD45RA^-CD90^+CD49f^{low}CD62L^-CD133^+$ (P5) populations in UCB $CD34^+$ cells before (fresh) and after culture in control, control medium supplemented with 1 ng/mL or 10 ng/mL human CXCL12 protein, Microniche. or Microniche supplemented with 0.04 nM or 0.08 nM LY2510924. **b** Percentages and absolute counts of live cells, P1, P2, P3, P4, and P5 populations before (fresh) and after 7 days *in vitro* culture assay of (**a**). Fresh, n = 4 technical replicates; other groups, n = 4 biological replicates. All data represent the means ± s.d.; unpaired two-tailed Students' t-test. Comparisons with control unless otherwise indicated; *$P < 0.05$; **$P < 0.01$; ***$P < 0.001$; ****$P < 0.0001$; ns = not significant.

Supplementary Table 1. Information on AA patient specimens. Proportion and absolute counts of total, $CD34^{+}CD38^{-}$, and $CD34^{+}CD38^{-}CD49f^{+}$ cells derived from AA patient BM MNCs after 7 days in culture are shown (*n* = 1 for AA1-5). # denotes absolute counts, % denotes percentage in live cells. For AA6 and AA8, *n* = 3 mice; For AA7, *n* = 6 (control) and 4 (Microniche) mice.

Specimen No.	Disease severity at diagnosis	Total cells		$CD34^{+}CD38^{-}$				$CD34^{+}CD38^{-}CD49f^{+}$				Assay	Additional info.
		Control	Microniche	Control		Microniche		Control		Microniche			
		# (×10^5)	# (×10^5)	%	# (×10^3)	%	# (×10^3)	%	# (×10^3)	%	# (×10^3)		
AA1	AA	1.72	1.56	0.16	0.27	0.82	1.28	0.12	0.20	0.66	1.03	*in vitro* culture	1×10^5 MNCs/well, 14 days *in vitro* culture
AA2	AA	0.70	0.92	1.02	0.71	1.50	1.38	0.40	0.28	1.47	1.36		
AA3	AA	0.90	1.01	0.23	0.21	3.36	3.39	0.11	0.10	3.26	3.30		
AA4	NSAA	1.10	1.30	0.11	0.12	0.39	0.51	-	-	-	-		
AA5	NSAA	1.50	1.40	0.14	0.21	0.74	1.04	-	-	-	-		
AA6	AA	-										NOG transplantation	2×10^5 MNCs/well, 12 days *in vitro* culture; i.v. 2×10^5 cultured cells
AA7	AA	-											2×10^5 MNCs/well, 12 days *in vitro* culture; intra-BM injection 5×10^4 cultured cells
AA8	AA	-											

Supplementary Table 2. Proportion and absolute counts of mice c-Kit$^+$ cells after culture *in vitro*. Values represent mean ± s.d. (n = 6 biological replicates), % denotes percentage in live cells unless otherwise specified, # denotes absolute counts. Comparisons with control, *p < 0.05, ****p < 0.0001 by unpaired two-tailed Student's *t*-test.

Subpopulations	Treatment			
	Control		Microniche	
	**%**	**# (×10^4)**	**%**	**# (×10^4)**
live cells		118 ± 21		112 ± 18
Lin$^-$	12.90 ± 2.81	15.26 ± 3.33	9.70 ± 1.23*	10.84 ± 1.37*
Lin$^-$c-Kit$^+$Sca-1$^-$	1.14 ± 0.04	1.35 ± 0.04	1.18 ± 0.23	1.31 ± 0.26
Lin$^-$c-Kit$^+$Sca-1$^+$	0.75 ± 0.04	0.89 ± 0.05	0.68 ± 0.08	0.76 ± 0.09
LKS$^+$CD34$^+$Flt3$^-$	0.56 ± 0.04	0.66 ± 0.04	0.16 ± 0.04****	0.18 ± 0.05****
LKS$^+$CD34$^-$Flt3$^-$	0.19 ± 0.03	0.22 ± 0.04	0.50 ± 0.07****	0.56 ± 0.08****
	**% in LKS$^+$**	**# (×10^2)**	**% in LKS$^+$**	**# (×10^2)**
LKS$^+$CD34$^-$CD150$^+$CD48$^-$	1.13 ± 0.13	1.01 ± 0.11	4.63 ± 1.12****	3.52 ± 0.85****
LKS$^+$CD34$^-$CD150$^+$CD48$^-$CD229$^-$	1.10 ± 0.13	0.98 ± 0.11	4.31 ± 0.94****	3.28 ± 0.72****

Supplementary Table 3. Proportion and absolute counts of phenotypically defined cell subsets before and after culture in a bioreactor in different conditions. Values represent mean ± s.d. (Fresh, *n* = 3 technical replicates; Microniche, *n* = 3 or 4 biological replicates), % denotes percentage in live cells, # denotes absolute counts, FC denotes fold change in absolute counts after culture. Comparisons with fresh, *p < 0.05, **p < 0.01, ***p < 0.001, ****p < 0.0001 by unpaired two-tailed Student's *t*-test.

**Treatment \ Subpopulations**		live cells		$CD34^+$			$CD34^+CD38^-$			$CD34^+CD38^-CD45RA^-CD90^+$			$CD34^+CD38^-CD45RA^-CD90^+CD49f^+$		
		**# (×10^8)**	**FC**	**%**	**# (×10^6)**	**FC**	**%**	**# (×10^6)**	**FC**	**%**	**# (×10^5)**	**FC**	**%**	**# (×10^4)**	**FC**
**Fresh**	day0	1.50 ± 0.00		2.47 ± 0.15	3.70 ± 0.23		0.74 ± 0.06	1.11 ± 0.09		0.06 ± 0.02	0.92 ± 0.29		0.01 ± 0.01	1.60 ± 0.76	
**Microniche**	day1	0.94 ± 0.01 ****	0.63	2.28 ± 0.23	2.14 ± 0.22 **	0.58	1.77 ± 0.20 **	1.66 ± 0.19 **	1.51	0.15 ± 0.01 **	1.45 ± 0.06 *	1.57	0.01 ± 0.00	1.32 ± 0.09	0.82
	day3	0.60 ± 0.04 ****	0.40	3.28 ± 1.58	1.96 ± 0.94 *	0.53	2.74 ± 0.28 ***	1.64 ± 0.17 **	1.48	0.45 ± 0.01 ****	2.71 ± 0.03 ***	2.93	0.05 ± 0.01 **	2.85 ± 0.50	1.78
	day5	0.55 ± 0.01 ****	0.36	4.82 ± 0.018 ****	2.63 ± 0.04 **	0.71	2.27 ± 0.06 ****	1.24 ± 0.03	1.12	1.17 ± 0.00 ****	6.37 ± 0.02 ****	6.89	0.05 ± 0.01 ***	2.86 ± 0.32	1.79
**Fresh**		0.66 ± 0.00		3.09 ± 0.09	2.04 ± 0.06		6.27 ± 0.13	4.14 ± 0.08		0.11 ± 0.02	0.72 ± 0.12		0.10 ± 0.02	6.87 ± 1.30	
**Microniche**	20 rpm	0.09 ± 0.00 ****	0.13	16.33 ± 0.67 ****	1.40 ± 0.06 ****	0.68	12.63 ± 0.22 ****	1.08 ± 0.02 ****	0.26	2.23 ± 0.04 ****	1.91 ± 0.03 ****	2.66	1.13 ± 0.05 ****	9.67 ± 0.40 **	1.41
	40 rpm	0.16 ± 0.00 ****	0.24	16.78 ± 0.10 ****	2.62 ± 0.01 ****	1.28	13.43 ± 0.30 ****	2.09 ± 0.05 ****	0.51	2.73 ± 0.12 ****	4.26 ± 0.18 ****	5.96	1.58 ± 0.08 ****	24.60 ± 1.21 ****	3.58
	80 rpm	0.13 ± 0.01 ****	0.20	14.80 ± 0.33 ****	1.94 ± 0.04 *	0.95	11.10 ± 0.29 ****	1.45 ± 0.04 ****	0.35	2.53 ± 0.06 ****	3.32 ± 0.08 ****	4.64	1.57 ± 0.02 ****	20.50 ± 0.25 ****	2.99
**Fresh**		1.00 ± 0.00		1.47 ± 0.08	1.47 ± 0.08		1.02 ± 0.06	1.02 ± 0.06		0.04 ± 0.00	0.42 ± 0.03		0.003 ± 0.00	0.35 ± 0.07	
**Microniche**	50 mg	0.28 ± 0.00 ****	0.28	32.95 ± 1.29 ****	9.14 ± 0.36 ****	6.24	23.80 ± 0.42 ****	6.60 ± 0.12 ****	6.47	3.15 ± 0.29 ****	8.74 ± 0.80 ****	20.77	2.26 ± 0.20 ****	62.75 ± 5.59 ****	179.29
	100 mg	0.18 ± 0.02 ****	0.18	32.80 ± 0.78 ****	6.04 ± 0.14 ****	4.12	22.70 ± 0.44 ****	4.18 ± 0.08 ****	4.10	2.47 ± 0.06 ****	4.54 ± 0.11 ****	10.80	1.76 ± 0.06 ****	32.41 ± 1.06 ****	92.61
	200 mg	0.17 ± 0.00 ****	0.17	31.30 ± 0.80 ****	5.43 ± 0.14 ****	3.71	20.93 ± 0.64 ****	3.63 ± 0.11 ****	3.56	3.23 ± 0.23 ****	5.60 ± 0.39 ****	13.30	2.51 ± 0.10 ****	43.55 ± 1.69 ****	124.44

Supplementary Table 4. Absolute counts of phenotypically defined cell subsets before (fresh) and after 3 days of culture in a bioreactor. Values represent mean ± s.d. (Fresh, *n* = 4 technical replicates; Microniche, *n* = 4 biological replicates), # denotes absolute counts. Comparisons with fresh, ****p < 0.0001 by unpaired two-tailed Student's *t*-test.

**Subpopulations**	**Treatment**		
	**Fresh**	**Microniche**	
	**# (×10^4)**	**# (×10^5)**	**Fold change**
live cells	1000 ± 0.00	277.4 ± 4.07 ****	0.28
$CD34^+$	14.65 ± 0.45	91.40 ± 3.57 ****	6.24
$CD34^+CD38^-$	10.20 ± 0.56	66.02 ± 1.18 ****	6.47
$CD34^+CD38^-CD45RA^-CD90^+$	0.42 ± 0.03	8.74 ± 0.80 ****	20.77
$CD34^+CD38^-CD45RA^-CD90^+CD49f^+$	0.03 ± 0.01	6.28 ± 0.56 ****	179.29
$CD34^+CD38^-CD45RA^-CD90^+CD49f^{low}$	0.03 ± 0.01	6.21 ± 0.54 ****	179.18
$CD34^+CD38^-CD45RA^-CD90^+CD49f^{low}CD62L^-CD133^+$	0.03 ± 0.01	3.81 ± 0.32 ****	123.23

Supplementary Table 5. Quantification of the expression of 20 cytokine-cytokine receptor genes after culture with Microniche (*n* = 4 biological replicates)**.**

Gene name	Fold change of gene expression
CXCL8	8.23 ± 0.36
CSF2RA	2.71 ± 0.28
CCL4L2	8.24 ± 0.41
CSF1R	3.38 ± 0.16
CX3CR1	5.55 ± 0.21
CCL4L1	7.25 ± 0.15
CXCL16	5.15 ± 0.29
CCL3L1	4.72 ± 0.30
CD40	2.89 ± 0.25
IL10RA	9.16 ± 0.67
CCL3L3	4.00 ± 0.30
CCL24	3.79 ± 0.28
CCL4	5.43 ± 1.05
CCL22	4.07 ± 0.27
IL21R	13.94 ± 1.45
FLT1	3.34 ± 0.68
IL13RA	4.43 ± 0.50
IL1R1	5.08 ± 1.20
IL1R2	9.66 ± 1.31
PDGFA	14.41 ± 2.84

Supplementary Table 6. Quantification of 40 chemokines from liquid suspension after culture with the control or Microniche (*n* = 3 biological replicates)**.**

Chemokines	Observed concentration (pg/mL)		Fold change
	Control	Microniche	
6Ckine (also known as CCL21)	96.80 ± 7.40	160.53 ± 11.24	1.66
BCA-1 (also known as CXCL13)	2.98 ± 0.52	55.03 ± 12.43	18.49
CTACK (also known as CCL27)	2.37 ± 0.87	10.20 ± 1.32	4.30
ENA-78 (also known as CXCL5)	381.26 ± 23.80	917.48 ± 207.27	2.41
Eotaxin (also known as CCL11)	8.92 ± 0.48	15.25 ± 1.40	1.71
Eotaxin-2 (also known as CCL24)	35.96 ± 14.93	273.86 ± 17.30	7.62
Eotaxin-3 (also known as CCL26)	4.19 ± 0.00	11.83 ± 1.87	2.82
Fractalkine (also known as CX3CL1)	14.44 ± 0.94	52.62 ± 6.52	3.64
GCP-2 (also known as CXCL6)	8.02 ± 1.12	16.61 ± 0.80	2.07
GM-CSF	42.18 ± 2.66	67.20 ± 6.13	1.59
Gro-alpha (also known as CXCL1)	55.33 ± 5.20	222.79 ± 39.74	4.03
Gro-beta (also known as CXCL2)	21.15 ± 3.40	87.20 ± 21.45	4.12
I-309 (also known as CCL1)	199.17 ± 16.24	911.63 ± 149.67	4.58
IFN-gamma	20.16 ± 0.81	46.04 ± 4.07	2.28
IL-1 beta	3.24 ± 0.22	8.73 ± 1.30	2.69
IL-2	2.97 ± 0.26	7.63 ± 0.68	2.57
IL-4	12.97 ± 0.46	24.98 ± 0.90	1.93
IL-6	37.01 ± 2.06	287.35 ± 166.53	7.76
IL-8 (also known as CXCL8)	809.44 ± 187.45	7051.01 ± 2227.65	8.71
IL-10	8.46 ± 0.91	19.19 ± 1.44	2.27
IL-16	35.41 ± 4.67	79.15 ± 12.14	2.24
IP-10 (also known as CXCL10)	96.98 ± 7.18	58.36 ± 1.55	0.60
I-TAC (also known as CXCL11)	0.51 ± 0.03	0.95 ± 0.12	1.87
MCP-1 (also known as CCL2)	6.70 ± 1.28	68.99 ± 12.32	10.30
MCP-2 (also known as CCL8)	0.97 ± 0.05	4.24 ± 0.20	4.37
MCP-3 (also known as CCL7)	42.61 ± 2.82	97.95 ± 8.64	2.30
MCP-4 (also known as CCL13)	7.49 ± 0.99	21.03 ± 1.55	2.81
MDC (also known as CCL22)	595.55 ± 84.36	1809.55 ± 539.01	3.04
MIF	8097.93 ± 378.42	17017.71 ± 2056.31	2.10
MIG (also known as CXCL9)	18.94 ± 1.31	39.61 ± 3.86	2.09
MIP-1 alpha (also known as CCL3)	10.77 ± 1.19	31.28 ± 1.57	2.90
MIP-1 delta (also known as CCL15)	6.41 ± 0.69	12.02 ± 1.70	1.87
MIP-3 alpha (also known as CCL20)	4.39 ± 0.10	16.02 ± 2.56	3.65
MIP-3 beta (also known as CCL19)	30.21 ± 4.90	66.22 ± 3.31	2.19
MPIF-1 (also known as CCL23)	112.87 ± 3.68	99.83 ± 0.70	0.88
SCYB16 (also known as CXCL16)	104.12 ± 1.02	182.05 ± 13.20	1.75
SDF-1 alpha+beta (also known as CXCL12)	79.84 ± 3.58	167.06 ± 12.53	2.09
TARC (also known as CCL17)	3.68 ± 0.54	13.07 ± 4.00	3.55
TECK (also known as CCL25)	74.74 ± 3.32	178.71 ± 20.68	2.39
TNF-alpha	17.30 ± 0.61	35.19 ± 5.46	2.03

Supplementary Table 7. Proportion and absolute counts of phenotypically defined cell subsets before and after culture with or without cytokines. Values represent mean ± s.d. (fresh *n* = 3 technical replicates; control and cytokines *n* = 4 biological replicates), % denotes percentage in live cells, # denotes absolute counts. Comparisons with fresh, *p < 0.05, **p < 0.01, ***p < 0.001, ****p < 0.0001 by unpaired two-tailed Student's *t*-test.

Subpopulations	Treatment					
	Fresh		Control		Cytokines	
	%	# (×10³)	%	# (×10³)	%	# (×10³)
live cells		200 ± 0.00		1601 ± 87****		1756 ± 134****
$CD34^+$	83.17 ± 0.49	166 ± 0.99	61.20 ± 2.64****	980 ± 42****	57.50 ± 3.04****	1013 ± 53****
$CD34^+CD38^-$	3.82 ± 0.27	7.63 ± 0.54	10.98 ± 1.03****	176 ± 16.49****	12.78 ± 0.30****	224 ± 5.24****
$CD34^+CD38^-CD45RA^-CD90^+$	0.72 ± 0.16	1.43 ± 0.32	1.02 ± 0.14*	16.38 ± 2.22****	1.06 ± 0.07*	18.63 ± 1.30****
$CD34^+CD38^-CD45RA^-CD49f^+$	0.02 ± 0.01	0.03 ± 0.01	0.03 ± 0.01	0.42 ± 0.10**	0.10 ± 0.04*	1.79 ± 0.70**
$CD34^+CD38^-CD45RA^-CD49f^{low}$	0.02 ± 0.01	0.03 ± 0.01	0.02 ± 0.01	0.40 ± 0.09***	0.09 ± 0.04*	1.63 ± 0.66**
$CD34^+CD38^-CD45RA^-CD49f^{low}CD62L^-CD133^+$	0.01 ± 0.00	0.02 ± 0.00	0.00 ± 0.00**	0.03 ± 0.01	0.01 ± 0.01	0.21 ± 0.18

Supplementary Table 8. List of antibodies and viability dye used in flow cytometry.

Application	Antibody or viability dye	Conjugate	Clone	Company	Catalog number
Analysis and cell sorting of human HSPCs expansion *in vitro*	CD34	APC	581	BD	555824
	CD34	FITC	561	BioLegend	343604
	CD38	PE-Cy7	HIT2	BD	560677
	CD45RA	APC-H7	HI100	BD	560674
	CD90	PerCP-Cy5.5	5E10	BD	561557
	CD90	APC	5E10	BD	559869
	CD49f	PE	GoH3	BD	555736
	CD49f	BV510	GoH3	BD	563271
	CD133	APC	W6B3C1	BD	566596
	CD62L	PE	DREG-56	BioLegend	304806
	DAPI	n/a	n/a	Sigma	MBD0015
Analysis of long-term culture of human UCB CD34$^+$ cells *in vitro*	CD34	APC	581	BD	555824
	CD38	PE-Cy7	HIT2	BD	560677
	CD45RA	APC-H7	HI100	BD	560674
	CD90	PerCP-Cy5.5	5E10	BD	561557
	CD49f	PE	GoH3	BD	555736
	CD71	FITC	OKT9	Invitrogen	11-0719-42
	CD110	BV421	1.6.1	BD	562672
	CD41a	BV510	HIP8	BD	563250
	DAPI	n/a	n/a	Sigma	MBD0015
Analysis of mouse HSPCs expansion *in vitro*	Ter119	PE-Cy7	TER-119	Invitrogen	25-5921-82
	Gr-1		RB6-8C5	Invitrogen	25-5931-82
	CD11b		M1/70	Invitrogen	25-0112-81
	B220		RA3-6B2	Invitrogen	25-0452-81
	CD3e		145-2C11	Invitrogen	25-0031-82
	CD4		GK1.5	Invitrogen	25-0041-82
	CD8a		53-6.7	Invitrogen	25-0081-81
	Sca-1	PerCP-Cy5.5	D7	Invitrogen	45-5981-82
	c-Kit	APC	2B8	Invitrogen	17- 1171-83
	CD34	FITC	RAM34	Invitrogen	11-0341-82
	CD16/32	BV421	93	BioLegend	101332
	Flt3	PE	A2F10	Invitrogen	12-1351-82
	Ter119	APC-Cy7	TER-119	BioLegend	116223
	Gr-1		RB6-8C5	BioLegend	108423
	CD11b		M1/70	BioLegend	101226
	B220		RA3-6B2	BioLegend	103224
	CD3e		145-2C11	BioLegend	100330
	CD4		RM4-5	BioLegend	100526
	CD8a		53-6.7	BioLegend	100714
	CD34	PE-Cy7	MEC14.7	BioLegend	119326
	CD150	BV421	TC15-12F12.2	BioLegend	115943
	CD48	FITC	HM48-1	Invitrogen	11-0481-85
	CD229	PE	Ly9ab3	BioLegend	122905
	DAPI	n/a	n/a	Sigma	MBD0015
	mCD45	PerCP-Cy5.5	HI30	BD	564105

Reconstitution analysis of HSPCs, myeloid cells, B and T lymphocytes, NK cells in BM	hCD45	FITC	HI30	BD	555482
	hCD33	APC-Cy7	P67.6	BioLegend	366614
	hCD19	PE	HIB19	BD	555413
	hCD3	BV650	SK7	BD	563999
	hCD56	BV786	B159	BD	740979
	hCD34	APC	581	BD	555824
	hCD38	PE-Cy7	HIT2	BD	560677
	hCD110	BV605	1.6.1	BD	743578
	hCD71	BV711	M-A712	BD	563767
	hCD41a	BV510	HIP8	BD	563250
	DAPI	n/a	n/a	Sigma	MBD0015
Reconstitution analysis of megakaryocytes and erythrocytes in BM	mCD45	APC-Cy7	30-F11	BD	557659
	mCD41	APC	MWReg30	BioLegend	133914
	mCD42d	PerCP-Cy5.5	1C2	BioLegend	148508
	mCD61	BV786	2C9.G2	BD	740867
	Ter119	PE-Cy7	TER-119	Invitrogen	25-5921-81
	mCD71	BV605	C2	BD	563013
	hCD45	FITC	HI30	BD	555482
	hCD41a	BV510	HIP8	BD	563250
	hCD42b	PE	HIP1	BD	555473
	hCD61	BV650	VI-PL2	BD	564172
	CD235a	Alexa Fluor 700	HIR2 (GA-R2)	Invitrogen	56-9987-42
	hCD71	BV711	M-A712	BD	563767
	DAPI	n/a	n/a	Sigma	MBD0015
BD Rhapsody Single-Cell Analysis	**Antibody-Oligo**	**SeqID**	**Clone**	**Company**	**Catalog number**
	CD34	AHS0061	581	BD	940021
	CD38	AHS0022	HIT2	BD	940013
	CD45RA	AHS0009	HI100	BD	940011
	CD90	AHS0045	5E10	BD	940032
	CD49f	AHS0119	GOH3	BD	940160

Supplementary Table 9. Primers for qRT-PCR.

Accession number	Primers	Forward (5'-3')	Reverse (5'-3')
NM_002982	CCL2	AAACTGAAGCTCGCACTCTC	AATCCTGAACCCACTTCTGC
NM_000576	IL1B	ATGGACAAGCTGAGGAAGATG	ACAAAGGACATGGAGAACACC
NM_000616	CD4	TCCTGCTTTTCATTGGGCTAG	CTGCTACATTCATCTGGTCCG
NM_000584	CXCL8	ATACTCCAAACCTTTCCACCC	GTTTCACTGGCATCTTCACTG
NM_005211	CSF1R	TGAAGGTGGCTGTGAAGATG	CGAGGTGGATGTTCTTATAGTCG
NM_001100812	CXCL16	CATCTTCATCCTCACCGCAG	AAGCCACAGTTTACCCTCAC
NM_001558	IL10RA	GCAGTGTGAACCTAGAGATCC	CTCTTTAGACCACATCCCCTTG
NM_002984	CCL4	ACCAATACCATGAAGCTCTGC	TTCAGTTCCAGGTCATACACG
NM_021798	IL21R	GTATGAAGAGCTGAAGGACGAG	CAGGGTCTTCGTAATCTGAGC
NM_001560	IL13RA1	CGCAATTCCACACTCTACATAAC	CTCCATCACTGAGAGGCTTTC
NM_004633	IL1R2	AAAATGACTCTGCTAGGACGG	TGAGATGAACGGCAGGAAAG
NM_006140	CSF2RA	TTAATGAACTGTACCTGGGCG	TTGCTGGGAGGGTTGAATC
NM_001337	CX3CR1	GTCTCTGGTAAAGTCTGAGCAG	ATGGCAAAGATGACGGAGTAG
NM_021006	CCL3L1	ATCACCTGCTCCCAATCATG	CACTGACGTATTTCTGGACCC
NM_001001437	CCL3L3	ATCACCTGCTCCCAATCATG	CACTGACGTATTTCTGGACCC
NM_002990	CCL22	GATTACGTCCGTTACCGTCTG	GATGGAGATCAGGGAATGCAG
NM_002019	FLT1	CTCAACTCCTGCCTTCTCTG	CCCCGACTCCTTACTTTTACTG
NM_000877	IL1R1	GCATCCTACACATACTTGGGC	CTAGCACTGGGTCATCTTCATC
NM_002607	PDGFA	CGTAGGGAGTGAGGATTCTTTG	CAGATCAGGAAGTTGGCGG
NM_001291468	CCL4L2	CCGCCTGCTGCTTTTCTTAC	TTGCCTACCACAGCTGGC
NM_207007	CCL4L1	AGCACTCTCAGCACCAATG	TTCAGTTCCAGGTCATACACG
NM_001250	CD40	AAGCGAATTCCTAGACACCTG	CGAAAGCAGATGACACATTGG
NM_002991	CCL24	TTCTGTTCCTTGGTGTCTGTG	TTCTGCTTGGCGTCCAG
NM_000609	CXCL12	GAGCCAACGTCAAGCATCTG	CGGGTCAATGCACACTTGTC
NM_001008540	CXCR4	GTCCATTCCTTTGCCTCTTTTG	ACTTGTCCGTCATGCTTCTC
NM_001123041	CCR2	ATTCTCCTGAACACCTTCCAG	TGACTTTCCTTTTCCACGACC
