# Supplementary material for: Expansion of human megakaryocyte-biased hematopoietic stem cells by biomimetic Microniche
Source: Nat Commun. 2023 Apr 18;14:2207. doi: 10.1038/s41467-023-37954-3 (PMC10113370; doi:10.1038/s41467-023-37954-3)
Supplement: Supplementary file 3 — Description of additional supplementary files [file 41467_2023_37954_MOESM3_ESM.pdf]

Description of additional supplementary files

Supplementary Dataset 1. Proportion and absolute counts of human hematopoietic cells before and after culture in vitro. For BM and PB MNC, n = 3 biological replicates; For UCB MNC, n = 4 technical replicates (Fresh) and n = 3 biological replicates (other groups); For UCB CD34+, n = 3 technical replicates(Fresh), n = 3 biological replicates (Microniche), and n = 4 biological replicates (other groups). Values represent mean ± s.d., % denotes percentage in live cells, # denotes absolute counts, FC denotes fold change in absolute counts after culture; compared with fresh, unpaired two-tailed Student's t-test, *p < 0.05, **p < 0.01, ***p < 0.001, ****p <0.0001 by unpaired two-tailed Student's *t*-test.

Supplementary Dataset 2. Levels of human engraftment in the BM of NOG mice 16 weeks post-transplantation with different day 0 equivalent cell doses of fresh UCB CD34+ cells or their progeny cultured with control or Microniche. If obvious positive cell subsets were observable, mice were considered to be successfully engrafted or reconstituted, regardless of the proportion. Data are in a colored grid.

Supplementary Dataset 3. Proportion and absolute counts of UCB $CD34^+$ cells after long-term culture *in vitro*. Values represent mean ± s.d., % denotes percentage in live cells, # denotes absolute counts. Fresh n = 4 technical replicates; Control, week 2 and week 6 n = 5 biological replicates, week 4 n = 6 biological replicates; UM171, week 2 and week 6 n = 5 biological replicates, week 4 n = 7 biological replicates; Microniche, week 2 and week 4 n = 5 biological replicates, week 6 n = 3 biological replicates.

Supplementary Dataset 4. Summary of human CD45 engraftment and multilineages reconstruction in primary NOG transplantation. Mk-HSC frequencies based on phenotypically defined subpopulations in fresh or cultured (7 days) UCB CD34+ cells were analyzed by ELDA.

Supplementary Dataset 5. Levels of human engraftment in the BM of NCG mice 16 weeks post-transplantation with different day 0 equivalent cell doses of fresh UCB MNCs or their progeny cultured with Microniche in dynamic bioreactor. We use no less than 0.01% mCD45-hCD45+ cells at week 16 as a standard of successful engraftment and no less than 0.01% hCD41a+ cells in mCD45-hCD45-mCD41-mCD42d-mCD62- population of recipient BM cells as a standard of successful Mk reconstruction. Data are in a colored grid.

Supplementary Dataset 6. Proportion and absolute counts of human hematopoietic cells before and after culture by different microcarriers *in vitro* (n=3). Values represent mean ± s.d., % denotes percentage in live cells, # denotes absolute counts, FC denotes fold change in absolute counts after culture; compared with fresh, unpaired two-tailed Student's t-test, *p < 0.05, **p < 0.01, ***p < 0.001, ****p < 0.0001.
