# Supplementary material for: Expansion of human megakaryocyte-biased hematopoietic stem cells by biomimetic Microniche
Source: Nat Commun. 2023 Apr 18;14:2207. doi: 10.1038/s41467-023-37954-3 (PMC10113370; doi:10.1038/s41467-023-37954-3)
Supplement: Supplementary file 10 — Reporting Summary [file 41467_2023_37954_MOESM10_ESM.pdf]

Corresponding author(s): Yingdai Gao

Last updated by author(s): Apr 3, 2023

Reporting Summary

Nature Portfolio wishes to improve the reproducibility of the work that we publish. This form provides structure for consistency and transparency in reporting. For further information on Nature Portfolio policies, see our Editorial Policies and the Editorial Policy Checklist.

Statistics

For all statistical analyses, confirm that the following items are present in the figure legend, table legend, main text, or Methods section.

n/a	Confirmed	
☐	☒	The exact sample size (*n*) for each experimental group/condition, given as a discrete number and unit of measurement
☐	☒	A statement on whether measurements were taken from distinct samples or whether the same sample was measured repeatedly
☐	☒	The statistical test(s) used AND whether they are one- or two-sided<br>*Only common tests should be described solely by name; describe more complex techniques in the Methods section.*
☒	☐	A description of all covariates tested
☒	☐	A description of any assumptions or corrections, such as tests of normality and adjustment for multiple comparisons
☐	☒	A full description of the statistical parameters including central tendency (e.g. means) or other basic estimates (e.g. regression coefficient) AND variation (e.g. standard deviation) or associated estimates of uncertainty (e.g. confidence intervals)
☐	☒	For null hypothesis testing, the test statistic (e.g. *F*, *t*, *r*) with confidence intervals, effect sizes, degrees of freedom and *P* value noted<br>*Give P values as exact values whenever suitable.*
☒	☐	For Bayesian analysis, information on the choice of priors and Markov chain Monte Carlo settings
☒	☐	For hierarchical and complex designs, identification of the appropriate level for tests and full reporting of outcomes
☐	☒	Estimates of effect sizes (e.g. Cohen's *d*, Pearson's *r*), indicating how they were calculated

Our web collection on statistics for biologists contains articles on many of the points above.

Software and code

Policy information about availability of computer code

Data collection	Flow Cytometry: BD FACSDiva Software (version 7.0 and version 8.0.1)<br>Confocal laser scanning microscopy (CLSM) and microscopically magnified colonies images: NIS-Elements F Software (version 4.00.06)<br>RT-PCR: Thermo Fisher Scientific QuantStudio™ Design & Analysis Software (version 1.4.3)
Data analysis	All statistical analyses were analysed by Graph Pad Prism (version 9.0) or Microsoft office Excel (version 2019).<br>Flow cytometry data were analysed by FlowJo_V10 Software (version 10.6.2).<br>Limiting dilution analysis was performed by ELDA (Last Modified: 24 October 2014)(http://bioinf.wehi.edu.au/software/elda/).<br>BD Rhapsody single cell RNA-seq data were analysed by BD SeqGeq Software (version 1.6.0).<br>10X Genomics single cell RNA-seq data were analysed by 10X Cell Ranger (version 2.1.0), Seurat R-package (version 4.0.1) and CellPhoneDB package (version 2.1.2).<br>RNA-seq data were analysed by DESeq2 R-package (version 1.10.1) and GSEA Software (version 4.1.0).<br>Fluorescence images were analysed by ImageJ (version 1.53c).<br>Cell morphology images were recorded and analyzed by NDP.view (version 2.9.22 RUO).

For manuscripts utilizing custom algorithms or software that are central to the research but not yet described in published literature, software must be made available to editors and reviewers. We strongly encourage code deposition in a community repository (e.g. GitHub). See the Nature Portfolio guidelines for submitting code & software for further information.

Data

Policy information about availability of data

All manuscripts must include a data availability statement. This statement should provide the following information, where applicable:

- Accession codes, unique identifiers, or web links for publicly available datasets
- A description of any restrictions on data availability
- For clinical datasets or third party data, please ensure that the statement adheres to our policy

Gene Set Enrichment analysis genesets (M9809, M14297, M4406) came from MSigDB collections of the GSEA geneset database (http://www.gsea-msigdb.org/gsea/msigdb/index.jsp). Canonical cell markers of interest in specific cell types (Supplementary Fig. 6f) came from the CellMarker database (http://bio-bigdata.hrbmu.edu.cn/CellMarker/). The main data supporting the results in this study are available within the paper and the Supplementary Information or from the corresponding authors upon reasonable request. The source data underlying the main and supplementary figures in this study are provided as a Source Data file. The raw sequence data reported in this paper have been deposited in the Genome Sequence Archive (Genomics, Proteomics & Bioinformatics 2021) in National Genomics Data Center (Nucleic Acids Res 2022), China National Center for Bioinformation / Beijing Institute of Genomics, Chinese Academy of Sciences (GSA-Human: HRA003310) that are publicly accessible at https://ngdc.cncb.ac.cn/gsa-human/browse/HRA003310.

Human research participants

Policy information about studies involving human research participants and Sex and Gender in Research.

Reporting on sex and gender	UCB/PB/BM cell samples were used regardless of sex and gender.
Population characteristics	3 adult PB samples and 58 UCB samples were collected from Shandong Qilu Stem Cell Engineering Co., Ltd. 3 BM samples from healthy donors and 8 BM samples from aplastic anemia patients were collected from the Blood Biobank of Institute of Hematology & Blood Diseases Hospital and frozen at -80°C. AA samples were reviewed by our chief physician (Jun Shi) to confirm patients' disease severity at diagnosis as described in Supplementary Table 1.
Recruitment	Fresh umbilical cord blood and adult peripheral blood were obtained from consenting donors by Shandong Cord Blood Hematopoietic Stem Cell Bank. Bone marrow from consenting healthy donors and aplastic anemia patients were obtained from Blood Biobank of Institute of Hematology & Blood Diseases Hospital. All samples are voluntary donations.
Ethics oversight	All donors agreed to experimental use of their MNCs after informed consent and our study was approved by the ethical committee (ethical review approval No: SBKT2020007-EC-2) in the Institute of Hematology and Blood Diseases Hospital, Chinese Academy of Medical Sciences.

Note that full information on the approval of the study protocol must also be provided in the manuscript.

Field-specific reporting

Please select the one below that is the best fit for your research. If you are not sure, read the appropriate sections before making your selection.

☒ Life sciences ☐ Behavioural & social sciences ☐ Ecological, evolutionary & environmental sciences

For a reference copy of the document with all sections, see nature.com/documents/nr-reporting-summary-flat.pdf

Life sciences study design

All studies must disclose on these points even when the disclosure is negative.

Sample size	No sample-size calculation was performed. In the scRNA-seq experiments, the number of analysed cells was selected according to standard protocols, on the basis of relevant previous studies, and to comply with technical requirements. BD Rhapsody single-cell analysis identified 7907 cells with 28465 features detected for the control sample and 8017 cells with 28465 features detected for the Microniche sample. 10X Genomics single-cell analysis identified 14830 cells for the control sample and 16744 cells for the Microniche sample. Sample size for the analysis of proportion of CD34-CD38-CD45RA-CD90+CD49f+ cells in UCB and PB MNCs was limited by quantity of cells from the samples (Fig. 1d, e and Supplementary Dataset 1). Sample size for the in vitro culture and xenograft experiments of AA specimens (Fig. 1g, h and Supplementary Table 1) was limited by availability of material from AA patients. Sample size for the long-term in vitro culture (Fig. 2a, b and Supplementary Dataset 3) was limited by decreasing cell numbers over time. Sample sizes for all experiments were sufficient to show the same trends between the three or more replicates performed for each experiment. Precise numbers and details on the experimental replicates are provided in the paper.
Data exclusions	Some cells from scRNA-seq were filtered out as part of a standard quality-check procedure. Mice that died during the rearing process before the end of the experiments were excluded for the final engraftment and reconstitution rate statistics.
Replication	All attempts at replication were successful. Findings were replicated as indicated in the figure legends.

Randomization	Samples and cells were randomly allocated into experimental groups. Mice were randomly and evenly assigned to different experimental groups by weight.
Blinding	Animal grouping and in vivo measurements were taken by investigators blinded to experimental groups. The investigators performing the in vitro experiments were not blinded to the experimental groups since they were the ones adding the different treatment. However, all experiments were performed in the same workflow as described in the methods to guarantee objective measurements and experiments were conducted independently by at least two investigators with similar results.

Reporting for specific materials, systems and methods

We require information from authors about some types of materials, experimental systems and methods used in many studies. Here, indicate whether each material, system or method listed is relevant to your study. If you are not sure if a list item applies to your research, read the appropriate section before selecting a response.

Materials & experimental systems

n/a	Involved in the study
☐	☒ Antibodies
☒	☐ Eukaryotic cell lines
☒	☐ Palaeontology and archaeology
☐	☒ Animals and other organisms
☒	☐ Clinical data
☒	☐ Dual use research of concern

Methods

n/a	Involved in the study
☒	☐ ChIP-seq
☐	☒ Flow cytometry
☒	☐ MRI-based neuroimaging

Antibodies

Antibodies used	The antibodies used for flow cytometry were as follows (provider, fluorophore, CatLog number, clone, dilution): anti-mouse CD45 (BD, PerCP-Cy5.5, Cat: 564105, Clone: HI30, 1:100; BD, APC-Cy7, Cat: 557659, Clone: 30-F11, 1:100); anti-mouse Gr-1 (Invitrogen, PE-Cy7, Cat: 25-5931-82, Clone: RB6-8C5, 1:100; Biolegend, APC-Cy7, Cat 108423, Clone: RB6-8C5, 1:100); anti-mouse CD11b (Invitrogen, PE-Cy7, Cat: 25-0112-81, Clone: M1/70, 1:100; Biolegend, APC-Cy7, Cat: 101226, Clone: M1/70, 1:100); anti-mouse B220 (Invitrogen, PE-Cy7, Cat: 25-0452-81, Clone: RA3-6B2, 1:100; Biolegend, APC-Cy7, Cat: 103224, Clone RA 3-6B2, 1:100); anti-mouse CD3e (Invitrogen, PE-Cy7, Cat: 25-0031-82, Clone: 145-2C11, 1:100; Biolegend, APC-Cy7, Cat: 100330, Clone: 145-2C11, 1:100); anti-mouse CD4 (Invitrogen, PE-Cy 7, Cat: 25-0041-82, Clone: GK1.5, 1:100; Biolegend, APC-Cy7, Cat: 100526, Clone: RM4-5, 1:100); anti-mouse CD8a (Invitrogen, PE-Cy7, Cat: 25-0081-81, Clone: 53-6.7, 1:100; Biolegend, APC-Cy7, Cat: 100714, Clone: 53-6.7, 1:100); anti-mouse Sca-1 (Invitrogen, PerCP-Cy5.5, Cat: 45-5981-82, Clone: D7, 1:100); anti-mouse c-Kit (Invitrogen , APC, Cat: 17-1171-83, Clone: 2B8, 1:100); anti-mouse CD34 (Invitrogen , FITC, Cat: 11-0341 -82, Clone: RAM34, 1:100; Biolegend, PE-Cy7, Cat : 119326, Clone: MEC14.7, 1:100); anti-mouse CD16/32 (Biolegend, BV421, Cat: 101332, Clone: 93, 1:100); anti-mouse Flt3 (Invitrogen , PE, Cat: 12-1351-82, Clone: A2F10, 1:100); anti-mouse CD150 (Biolegend, BV421, Cat: 115943, Clone: TC15-12F12.2, 1:100); anti-mouse CD48 (Invitrogen, FITC, Cat: 11-0481-85, Clone: HM48-1, 1:100); anti-mouse CD229 (Biolegend, PE, Cat: 122905, Clone: Ly9ab3, 1:100); anti-mouse CD41 (Biolegend, APC, Cat: 133914, Clone: MWReg30, 1:100); anti-mouse CD42d (Biolegend, PerCP-Cy5.5, Cat: 148508, Clone: 1C2, 1:100); anti-mouse CD61 (BD, BV786, Cat: 740867, Clone: 2C9.G2, 1:100); anti -mouse Ter119 (Invitrogen, PE-Cy7, Cat: 25-5921-81, Clone: TER-119, 1:100; Biolegend, APC-Cy7, Cat: 116223 , Clone: TER-119, 1:100); anti-mouse CD71 (BD, BV605 , Cat: 563013, Clone: C2, 1:100); anti-human CD34 (BD, APC, Cat: 555824, Clone: 581, 1:100; Biolegend, FITC, Cat: 343604, Clone: 561, 1:100); anti-human CD38 (BD, PE-Cy7, Cat: 560677, Clone: HIT2, 1:100); anti-human CD45RA (BD, APC-H7, Cat: 560674, Clone: HI100, 1:100); anti-human CD90 (BD, PerCP-Cy5.5 , Cat: 561557, Clone: 5E10, 1:100; BD, APC, Cat: 559869, Clone: 5E10, 1:100); anti-human CD49f (BD, PE, Cat: 555736, Clone: GoH3, 1:100; BD, BV510, Cat: 563271, Clone: GoH3, 1:100); anti-human CD133 (BD, APC, Cat: 566596, Clone: W6B3C1, 1:100); anti-human CD62L (Biolegend, PE, Cat: 304806, Clone: DREG-56, 1:100); anti - human CD71 (Invitrogen, FITC, Cat: 11-0719-42, Clone: OKT9, 1:100; BD, BV711, Cat: 563767, Clone: M-A712, 1:100) ; anti-human CD110 (BD, BV421, Cat: 562672, Clone: 1.6.1, 1:100; BD, BV605 , Cat: 743578, Clone: 1.6.1, 1:100); anti-human CD41a (BD, BV510 , Cat: 563250, Clone: HIP8, 1:100); anti-human CD42b (BD, PE, Cat: 555473, Clone: HIP1, 1:100); anti-human CD61 (BD, BV650 , Cat: 564172, Clone: VI-PL2, 1:100); anti-human CD45 (BD, FITC, Cat: 555482, Clone: HI30, 1:100); anti-human CD33 (Biolegend, APC-Cy7, Cat: 366614, Clone: P67.6, 1:100); anti-human CD19 (BD, PE, Cat: 555413, Clone: HIB19, 1:100); anti-human CD3 (BD, BV650, Cat: 563999, Clone: SK7, 1:100); anti-human CD56 (BD , BV786, Cat: 740979, Clone: B159, 1:100); anti-human CD235a (Invitrogen, Alexa Fluor 700, Cat: 56-9987-42, Clone: HIR2 (GA-R2), 1:100). Dead cells were excluded by DAPI (Sigma, MBD0015, 1:1000). The Oligo antibodies used for BD Rhapsody Single-Cell Analysis were as follows: oligo mouse anti-human CD34 (BD, SeqlD: AHS0061, Cat: 940021, Clone: 581, 1:25); oligo mouse anti-human CD38 (BD, SeqlD: AHS0022, Cat: 940013, Clone: HIT2, 1:25); oligo mouse anti-human CD45RA (BD, SeqlD: AHS0009, Cat : 940011, Clone: HI100, 1:25); oligo mouse anti-human CD90 (BD, SeqlD: AHS0045, Cat: 940032, Clone: 5E10, 1:25); oligo mouse anti-human CD49f (BD , SeqlD: AHS0119, Cat: 940160, Clone: GOH3, 1:25).
Validation	Validations were conducted by the respective manufacturer, as noted on the antibody specification sheet.<br>https://www.bdbiosciences.com/en-eu/products/reagents/flow-cytometry-reagents/research-reagents/single-color-antibodies-ruo/percp-cy-5-5-mouse-anti-human-cd45.564105<br>https://www.bdbiosciences.com/en-eu/products/reagents/flow-cytometry-reagents/research-reagents/single-color-antibodies-ruo/apc-cy-7-rat-anti-mouse-cd45.557659<br>https://www.thermofisher.cn/cn/zh/antibody/product/Ly-6G-Ly-6C-Antibody-clone-RB6-8C5-Monoclonal/25-5931-82<br>https://www.biolegend.com/en-us/products/apc-cyanine7-anti-mouse-ly-6gly-6c-gr-1-antibody-3935<br>https://www.thermofisher.cn/cn/zh/antibody/product/CD11b-Antibody-clone-M1-70-Monoclonal/25-0112-81<br>https://www.biolegend.com/en-us/products/apc-cyanine7-anti-mouse-human-cd11b-antibody-3930<br>https://www.thermofisher.cn/cn/zh/antibody/product/CD45R-B220-Antibody-clone-RA3-6B2-Monoclonal/25-0452-82

https://www.biolegend.com/en-us/products/apc-cyanine7-anti-mouse-human-cd45r-b220-antibody-1938
https://www.thermofisher.cn/cn/zh/antibody/product/CD3e-Antibody-clone-145-2C11-Monoclonal/25-0031-82
https://www.biolegend.com/en-us/products/apc-cyanine7-anti-mouse-cd3epsilon-antibody-6070
https://www.thermofisher.cn/cn/zh/antibody/product/CD4-Antibody-clone-GK1-5-Monoclonal/25-0041-82
https://www.biolegend.com/en-us/products/apc-cyanine7-anti-mouse-cd4-antibody-1937
https://www.thermofisher.cn/cn/zh/antibody/product/CD8a-Antibody-clone-53-6-7-Monoclonal/25-0081-81
https://www.biolegend.com/en-us/products/apc-cyanine7-anti-mouse-cd8a-antibody-2269
https://www.thermofisher.cn/cn/zh/antibody/product/Ly-6A-E-Sca-1-Antibody-clone-D7-Monoclonal/45-5981-82
https://www.thermofisher.cn/cn/zh/antibody/product/CD117-c-Kit-Antibody-clone-2B8-Monoclonal/17-1171-83
https://www.thermofisher.cn/cn/zh/antibody/product/CD34-Antibody-clone-RAM34-Monoclonal/11-0341-82
https://www.biolegend.com/en-us/products/pe-cyanine7-anti-mouse-cd34-antibody-14817
https://www.biolegend.com/en-us/products/brilliant-violet-421-anti-mouse-cd16-32-antibody-8598
https://www.thermofisher.cn/cn/zh/antibody/product/CD135-Flt3-Antibody-clone-A2F10-Monoclonal/12-1351-82
https://www.biolegend.com/en-us/products/brilliant-violet-421-anti-mouse-cd150-slam-antibody-7162
https://www.thermofisher.cn/cn/zh/antibody/product/CD48-Antibody-clone-HM48-1-Monoclonal/11-0481-85
https://www.biolegend.com/en-us/products/pe-anti-mouse-cd229-ly-9-antibody-3995
https://www.biolegend.com/en-us/products/apc-anti-mouse-cd41-antibody-7592
https://www.biolegend.com/en-us/products/percp-cyanine5-5-anti-mouse-rat-cd42d-antibody-10737
https://www.bdbiosciences.com/en-eu/products/reagents/flow-cytometry-reagents/research-reagents/single-color-antibodies-ruo/bv786-hamster-anti-mouse-cd61.740867
https://www.thermofisher.cn/cn/zh/antibody/product/TER-119-Antibody-clone-TER-119-Monoclonal/25-5921-81
https://www.biolegend.com/en-us/products/apc-cyanine7-anti-mouse-ter-119-erythroid-cells-antibody-3905
https://www.bdbiosciences.com/en-eu/products/reagents/flow-cytometry-reagents/research-reagents/single-color-antibodies-ruo/bv605-rat-anti-mouse-cd71.563013
https://www.bdbiosciences.com/en-eu/products/reagents/flow-cytometry-reagents/research-reagents/single-color-antibodies-ruo/apc-mouse-anti-human-cd34.555824
https://www.biolegend.com/en-us/products/fitc-anti-human-cd34-antibody-6035
https://www.bdbiosciences.com/en-eu/products/reagents/flow-cytometry-reagents/research-reagents/single-color-antibodies-ruo/pe-cy-7-mouse-anti-human-cd38.560677
https://www.bdbiosciences.com/en-eu/products/reagents/flow-cytometry-reagents/research-reagents/single-color-antibodies-ruo/apc-h7-mouse-anti-human-cd45ra.560674
https://www.bdbiosciences.com/en-eu/products/reagents/flow-cytometry-reagents/research-reagents/single-color-antibodies-ruo/percp-cy-5-5-mouse-anti-human-cd90.561557
https://www.bdbiosciences.com/en-eu/products/reagents/flow-cytometry-reagents/research-reagents/single-color-antibodies-ruo/apc-mouse-anti-human-cd90.559869
https://www.bdbiosciences.com/en-eu/products/reagents/flow-cytometry-reagents/research-reagents/single-color-antibodies-ruo/pe-rat-anti-human-cd49f.555736
https://www.bdbiosciences.com/en-eu/products/reagents/flow-cytometry-reagents/research-reagents/single-color-antibodies-ruo/bv510-rat-anti-human-cd49f.563271
https://www.bdbiosciences.com/en-eu/products/reagents/flow-cytometry-reagents/research-reagents/single-color-antibodies-ruo/apc-mouse-anti-human-cd133.566596
https://www.biolegend.com/en-us/products/pe-anti-human-cd62l-antibody-653
https://www.thermofisher.cn/cn/zh/antibody/product/CD71-Transferrin-Receptor-Antibody-clone-OKT9-OKT-9-Monoclonal/11-0719-42
https://www.bdbiosciences.com/en-eu/products/reagents/flow-cytometry-reagents/research-reagents/single-color-antibodies-ruo/bv711-mouse-anti-human-cd71.563767
https://www.bdbiosciences.com/en-eu/products/reagents/flow-cytometry-reagents/research-reagents/single-color-antibodies-ruo/bv421-mouse-anti-human-cd110.562672
https://www.bdbiosciences.com/en-eu/products/reagents/flow-cytometry-reagents/research-reagents/single-color-antibodies-ruo/bv605-mouse-anti-human-cd110.743578
https://www.bdbiosciences.com/en-eu/products/reagents/flow-cytometry-reagents/research-reagents/single-color-antibodies-ruo/bv510-mouse-anti-human-cd41a.563250
https://www.bdbiosciences.com/en-eu/products/reagents/flow-cytometry-reagents/research-reagents/single-color-antibodies-ruo/pe-mouse-anti-human-cd42b.555473
https://www.bdbiosciences.com/en-eu/products/reagents/flow-cytometry-reagents/research-reagents/single-color-antibodies-ruo/bv650-mouse-anti-human-cd61.564172
https://www.bdbiosciences.com/en-eu/products/reagents/flow-cytometry-reagents/research-reagents/single-color-antibodies-ruo/fitc-mouse-anti-human-cd45.555482
https://www.biolegend.com/en-us/products/apc-cyanine7-anti-human-cd33-antibody-12256
https://www.bdbiosciences.com/en-eu/products/reagents/flow-cytometry-reagents/research-reagents/single-color-antibodies-ruo/pe-mouse-anti-human-cd19.555413
https://www.bdbiosciences.com/en-eu/products/reagents/flow-cytometry-reagents/research-reagents/single-color-antibodies-ruo/bv650-mouse-anti-human-cd3.563999
https://www.bdbiosciences.com/en-eu/products/reagents/flow-cytometry-reagents/research-reagents/single-color-antibodies-ruo/bv786-mouse-anti-human-cd56-ncam-1.740979
https://www.thermofisher.cn/cn/zh/antibody/product/CD235a-Glycophorin-A-Antibody-clone-HIR2-GA-R2-Monoclonal/56-9987-42
https://www.sigmaaldrich.cn/CN/zh/product/sigma/mbd0015
https://www.bdbiosciences.com/en-eu/products/reagents/single-cell-multiomics-reagents/bd-abseq-assay/oligo-mouse-anti-human-cd34.940021
https://www.bdbiosciences.com/en-eu/products/reagents/single-cell-multiomics-reagents/bd-abseq-assay/oligo-mouse-anti-human-cd38.940013
https://www.bdbiosciences.com/en-eu/products/reagents/single-cell-multiomics-reagents/bd-abseq-assay/oligo-mouse-anti-human-cd45ra.940011
https://www.bdbiosciences.com/en-eu/products/reagents/single-cell-multiomics-reagents/bd-abseq-assay/oligo-mouse-anti-human-cd90.940032

https://www.bdbiosciences.com/en-eu/products/reagents/single-cell-multiomics-reagents/bd-abseq-assay/oligo-rat-anti-human-cd49f.940160

Animals and other research organisms

Policy information about studies involving animals; ARRIVE guidelines recommended for reporting animal research, and Sex and Gender in Research

Laboratory animals	NOD/Shi-scid/IL-2Rγnull (NOG) mice (female, 6-7 weeks old) were purchased from Charles River Laboratories. NOD/ShiLtJGpt-Prkdc(em26Cd52)Il2rg(em26Cd22)/Gpt (NCG) mice (female, 6-7 weeks) were purchased from GemPharmatech Co., LTD. C57BL/6J mice (female, 8-10 weeks old) were purchased from Beijing HFK Bioscience Co., LTD. All mice were housed under specific-pathogen-free (SPF) conditions, light/dark cycle: 12h/12h, temperature: 18-23°C, humidity: 40-60%, with free access to food and water.
Wild animals	The study did not involve wild animals.
Reporting on sex	Female mice were used in this study.
Field-collected samples	The study did not involve samples collected from the field.
Ethics oversight	The study was performed with the approval of the Animal Care and Use Committee of State Key Laboratory of Experimental Hematology, Institute of Hematology and Blood Diseases Hospital. All mouse experimental procedures were performed in accordance with the Regulations for the Administration of Affairs Concerning Experimental Animals approved by the State Council of the People's Republic of China.

Note that full information on the approval of the study protocol must also be provided in the manuscript.

Flow Cytometry

Plots

Confirm that:

☒ The axis labels state the marker and fluorochrome used (e.g. CD4-FITC).

☒ The axis scales are clearly visible. Include numbers along axes only for bottom left plot of group (a 'group' is an analysis of identical markers).

☒ All plots are contour plots with outliers or pseudocolor plots.

☒ A numerical value for number of cells or percentage (with statistics) is provided.

Methodology

Sample preparation	Cells were collected and the live cells were counted using trypan blue and an automated cell counter (Bio-Rad, TC20). For in vitro culture, cells after cultured with Cytopore1, Cytodex3, or Microniche were resuspended via gentle pipetting, and the cells were filtered with a 30 μm sterile nylon membrane. Mouse BM cells were flushed out from ilia, femurs, and tibias into PBS with 2 mM EDTA (Sigma-Aldrich), and were filtered with a 30 μm sterile nylon membrane. Then, the single-cell suspensions were stained with fluorescent-labeled antibodies in PBS supplemented with 2% FBS at 4 centigrade for 30 minutes, after which the stained cells were washed once with PBS. Details are provided in Methods.
Instrument	BD FACSCanto II, LSRII, FACSAriaIII.
Software	FlowJo_V10 Software (version 10.6.2).
Cell population abundance	The purity of FACSAriaIII sorted sample is above 99% as confirmed by re-examing the sorted samples under flow cytometer.
Gating strategy	First, cells were gated on FSC-A/SSC-A; and second, single cells were gated on FSC-H/FSC-A or SSC-H/SSC-A. Positive signal was gated based on unstained cells as the negative control. The gating strategy is graphically represented in Fig. 2c and Supplementary Fig. 1e, 3d.

☒ Tick this box to confirm that a figure exemplifying the gating strategy is provided in the Supplementary Information.
